# Supplementary material for: Cas9 is mostly orthogonal to human systems of DNA break sensing and repair
Source: PLoS One. 2023 Nov 29;18(11):e0294683. doi: 10.1371/journal.pone.0294683 (PMC10686484; doi:10.1371/journal.pone.0294683)
Supplement: S1 Table — (DOCX) [file pone.0294683.s001.docx]

**S1 Table.** **Oligonucleotides used in this study.**

| **Name** | **Sequence, 5′→3′** |
| --- | --- |
| ***Cas9 activity assay*** | |
| DNA1 | CTGATAACTCAATTTGTAAAAAATGGTACTGAGCA |
| DNA2 | TGCTCAGTACCATTTTTTACAAATTGAGTTATCAG |
| ***pLK1 plasmid construction*** | |
| pLK1.TOP | TCGAGATAACTCAATTTGTAAAAAATGGTAG |
| pLK1.BTM | AATTCTACCATTTTTTACAAATTGAGTTATC |
| ***Guide RNA cloning into pX458*** | |
| pX458.H4.TOP | CACCTGTCTGGGGACACGTCTCCA |
| pX458.H4.BTM | AAACTGGAGACGTGTCCCCAGACA |
| pX458.H6.TOP | CACCGAATGAAAATGCGGTTCTTG |
| pX458.H6.BTM | AAACCAAGAACCGCATTTTCATTC |
| pX458.H9.TOP | CACCCCGTCACTGAGACAGTGCGC |
| pX458.H9.BTM | AAACGCGCACTGTCTCAGTGACGG |
| ***TIDE analysis*** | |
| TIDE.H4.FWD | TGGCAGGGCTGGTCTTTCTCTGGCA |
| TIDE.H4.REV | AGTCCCGAGCCAAAGCCGAGTGACA |
| TIDE.H6.FWD | TTCTCCTTTCAGATATGGCTGG |
| TIDE.H6.REV | AAGAGATGAATGTATGGGTATGGCT |
| TIDE.H9.FWD | GCTGCCTCTGTTCTTCACCT |
| TIDE.H9.REV | GGTTAAAATGTCACCAGGGTCC |
