# Supplementary material for: Cas9 is mostly orthogonal to human systems of DNA break sensing and repair
Source: PLoS One. 2023 Nov 29;18(11):e0294683. doi: 10.1371/journal.pone.0294683 (PMC10686484; doi:10.1371/journal.pone.0294683)
Supplement: S2 Table — (DOCX) [file pone.0294683.s002.docx]

**S2 Table.** **PCR protocol for genomic targets amplification.**

| **Temperature, °C** | **Time** | **Number of cycles** |
| --- | --- | --- |
| 95 | 3 min | 1 |
| 95 | 30 s | 35 |
| 72 (H4)  62 (H6, H9) | 30 s |  |
| 72 | 30 s |  |
| 72 | 5 min | 1 |
| 4 | ∞ | 1 |
