# Supplementary figures and images for: Cas9 is mostly orthogonal to human systems of DNA break sensing and repair
Source: PLoS One. 2023 Nov 29;18(11):e0294683. doi: 10.1371/journal.pone.0294683 (PMC10686484; doi:10.1371/journal.pone.0294683)

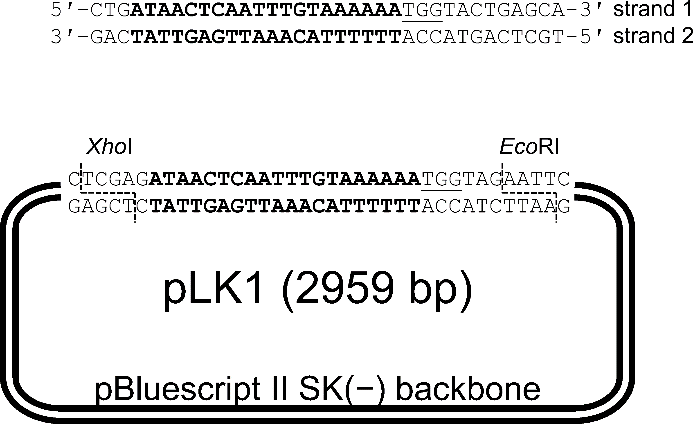


**S1 Fig**. **Substrates used in the Cas9 activity assays.**

Supplement: S1 Fig — (DOCX) [file pone.0294683.s003.docx]
