# Supplementary material for: Cas9 is mostly orthogonal to human systems of DNA break sensing and repair
Source: PLoS One. 2023 Nov 29;18(11):e0294683. doi: 10.1371/journal.pone.0294683 (PMC10686484; doi:10.1371/journal.pone.0294683)
Supplement: S2 Fig — (DOCX) [file pone.0294683.s004.docx]

**
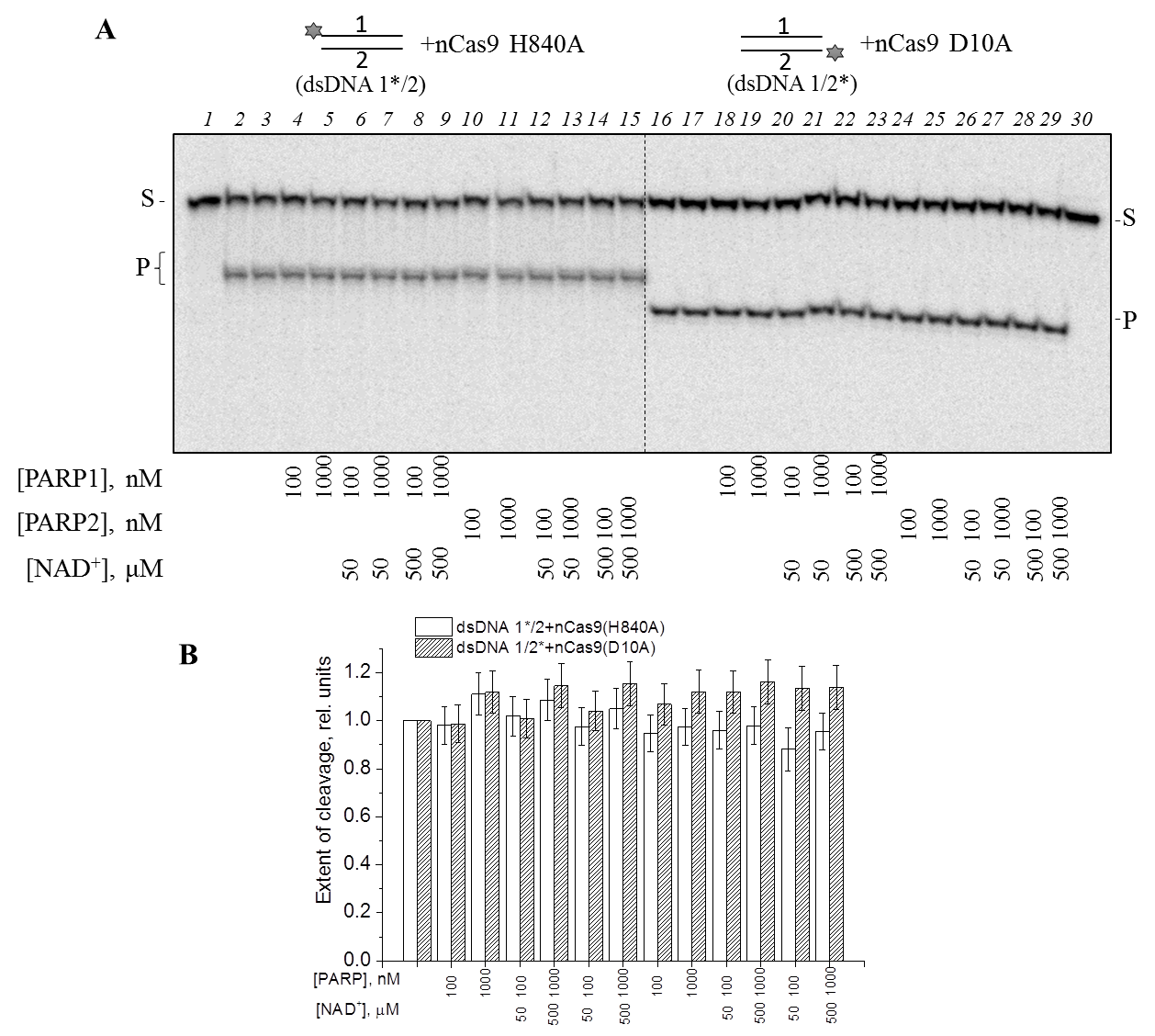
S2 Fig. Cleavage of the oligonucleotide substrate by Cas9 nickase mutants in the presence of PARP1 and PARP2.** The endonuclease activity was tested by incubation of nCas9 H840A/sgRNA or nCas9 D10A/sdRNA (20 nM) with dsDNA 1*/2 or dsDNA 1/2* (10 nM) at 37°C for 30 min, in the absence (lanes 2, 3 and 16, 17) and presence (lanes 4–15 and 18–29) of PARP1/PARP2, without or with NAD^+^. The reaction products were separated in a denaturing 20% PAG as shown in panel A. Bar charts (B) show the extent of dsDNA 1/2 cleavage induced by nCas9/sgRNA in the absence or presence of PARP1/PARP2 normalized to that in the absence of PARPs (the mean ± SD, n = 3). The 5’-^32^P-labelled strand in dsDNA is asterisked.
