# Supplementary material for: Cas9 is mostly orthogonal to human systems of DNA break sensing and repair
Source: PLoS One. 2023 Nov 29;18(11):e0294683. doi: 10.1371/journal.pone.0294683 (PMC10686484; doi:10.1371/journal.pone.0294683)
Supplement: S3 Fig — (DOCX) [file pone.0294683.s005.docx]

**
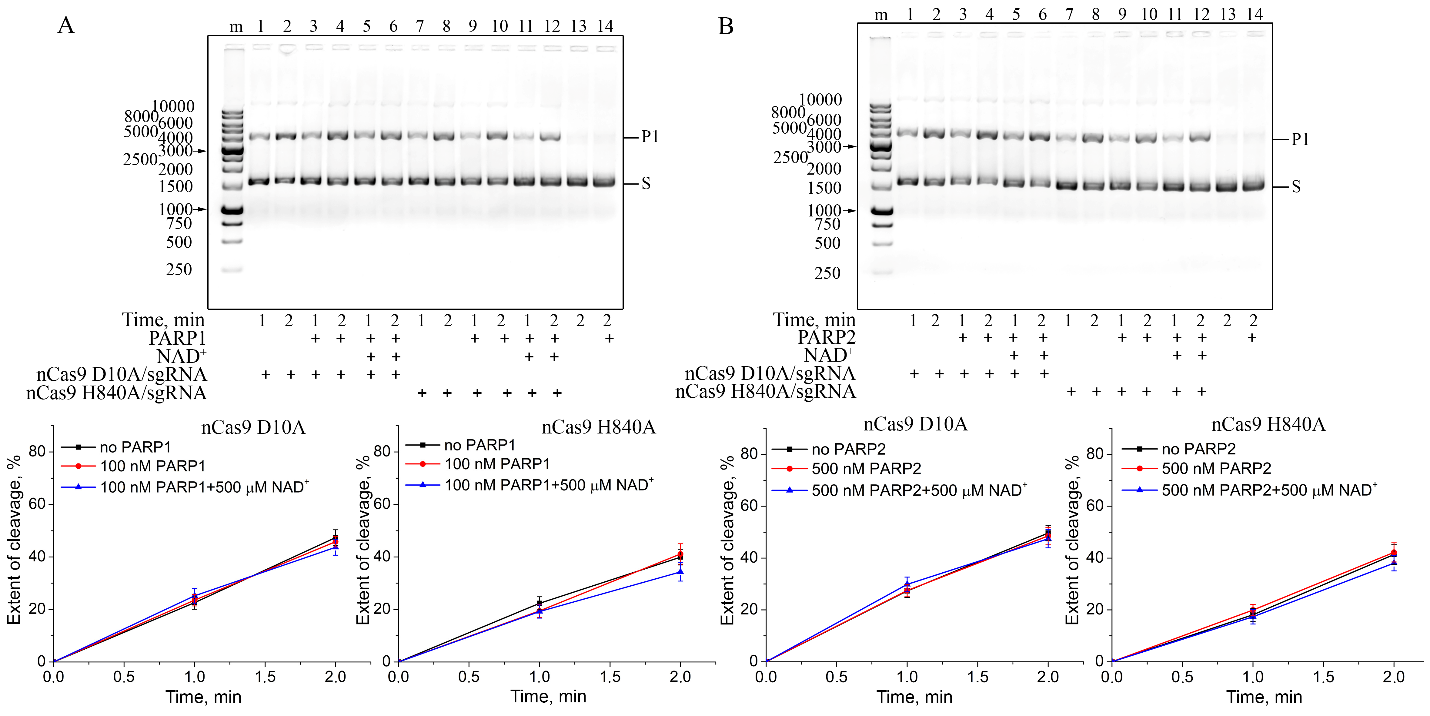
S3 Fig. Cleavage of the plasmid substrate by Cas9 nickase mutants in the presence of PARP1 and PARP2.** The nCas9 D10A/sgRNA or nCas9 H840A/sgRNA complex (10 nM) was incubated with pLK1 DNA (10 ng/µl) at 37°C, in the absence (lanes 1, 2 and 7, 8) and presence (lanes 3–6 and 9–12) of PARP1 (100 nM) or PARP2 (500 nM) supplemented with 500 µM NAD^+^ or not. The single-strand cleavage product (Р1) was separated from the substrate (S) by electrophoresis in 1% GelRed stained agarose gel. The plots show the accumulation of cleavage product under the indicated conditions (the mean ± SD, n = 3).
