# Supplementary material for: Cas9 is mostly orthogonal to human systems of DNA break sensing and repair
Source: PLoS One. 2023 Nov 29;18(11):e0294683. doi: 10.1371/journal.pone.0294683 (PMC10686484; doi:10.1371/journal.pone.0294683)
Supplement: S4 Fig — (DOCX) [file pone.0294683.s006.docx]

**S4 Fig. Binding of Cas9 and its mutant forms to the dsDNA substrate of Cas9**. The EMSA analysis of dsDNA 1/2* binding to Cas9 and Cas9/sgRNA (A), Cas9/sgRNA and nCas9 D10A/sgRNA (B), nCas9 H840A/sgRNA and dCas9/sgRNA (C). The reaction mixtures containing 10 nM dsDNA 1/2* and Cas9/Cas9 mutant (free or in the complex with sgRNA) at varied concentrations were incubated in the absence of Mg^2+^ at 4°C for 30 min and separated in a native 5% PAG. The apparent K_d_ values of the complexes (the mean ± SD, *n* = 3) are presented in Table (D).
