# Supplementary material for: Cas9 is mostly orthogonal to human systems of DNA break sensing and repair
Source: PLoS One. 2023 Nov 29;18(11):e0294683. doi: 10.1371/journal.pone.0294683 (PMC10686484; doi:10.1371/journal.pone.0294683)
Supplement: S5 Fig — (DOCX) [file pone.0294683.s007.docx]

**

S5 Fig. Binding of PARP1 and PARP2 to the dsDNA substrate of Cas9**. The reaction mixtures containing 10 nM dsDNA 1/2* and PARP1 (A)/PARP2 (B) at the indicated concentrations were incubated in the absence (lanes 1–9) or presence (lanes 10–17) of 500 μM NAD^+^ at 4°C for 30 min (in the absence of Mg^2+^) and separated in a native 5% PAG. The apparent K_d_ values of the complexes (the mean ± SD, *n* = 3) are presented in Table (C).
