# Supplementary material for: Cas9 is mostly orthogonal to human systems of DNA break sensing and repair
Source: PLoS One. 2023 Nov 29;18(11):e0294683. doi: 10.1371/journal.pone.0294683 (PMC10686484; doi:10.1371/journal.pone.0294683)
Supplement: S6 Fig — (DOCX) [file pone.0294683.s008.docx]

**

S6 Fig. Binding of dCas9/sgRNA complex, PARP1 and PARP2 to plasmid DNA**. The pLK1 plasmid DNA (10 ng/µl) was incubated in the absence and presence of increasing concentrations of dCas9/sgRNA, PARP1 or PARP2. EMSA for the protein-DNA complexes was performed by electrophoresis in 1% GelRed stained agarose gel.
