# Supplementary material for: Cas9 is mostly orthogonal to human systems of DNA break sensing and repair
Source: PLoS One. 2023 Nov 29;18(11):e0294683. doi: 10.1371/journal.pone.0294683 (PMC10686484; doi:10.1371/journal.pone.0294683)
Supplement: S7 Fig — (DOCX) [file pone.0294683.s009.docx]

**

S7 Fig. Protection of Cas9-generated dsDNA1/2 cleavage product from degradation in cell extracts.** Cas9/sgRNA (10 nM) was incubated with dsDNA1*/2 or dsDNA1/2* (10 nM), HEK293 or HEK293 *PARP1*^−/−^ cell extracts and 500 µM NAD^+^ as indicated. Cas9/sgRNA was pre-incubated with the substrate on ice for 60 min without Mg^2+^, then the reaction mixtures were supplemented with cell extracts and 10 mM MgCl_2_ and further incubated at 37°C for 30 min. (A) Representative gel images. (B) Relative yield of Cas9-cleaved DNA (P_Cas9_) in the presence of cell extracts normalized to that in the absence of the extracts.
