# Supplementary material for: Cas9 is mostly orthogonal to human systems of DNA break sensing and repair
Source: PLoS One. 2023 Nov 29;18(11):e0294683. doi: 10.1371/journal.pone.0294683 (PMC10686484; doi:10.1371/journal.pone.0294683)
Supplement: S8 Fig — (DOCX) [file pone.0294683.s010.docx]

**
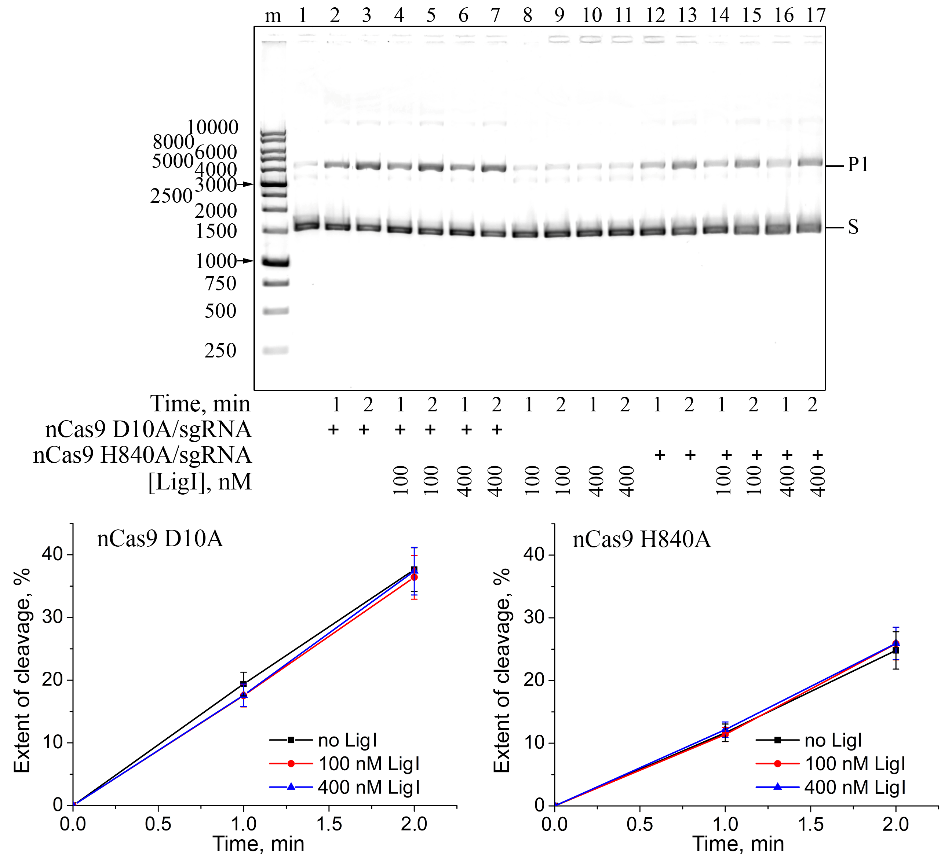
S8 Fig. Effects of DNA ligase I on the nickase activity of Cas9.** (A) nCas9 D10A/sgRNA (10 nМ) or nCas9 H840A/sgRNA (10 nM) was incubated with pLK1 DNA (10 ng/µl) at 37°C, in the absence and presence (+) of LigI (100 or 400 nM) (as detailed in Methods). The product (Р1) was separated from the substrate (S) by electrophoresis in 1% GelRed stained agarose gel. The plots show accumulation of the cleavage product in the absence and presence of LigI (the mean ± SD, n = 3).
