# Supplementary material for: Cas9 is mostly orthogonal to human systems of DNA break sensing and repair
Source: PLoS One. 2023 Nov 29;18(11):e0294683. doi: 10.1371/journal.pone.0294683 (PMC10686484; doi:10.1371/journal.pone.0294683)
Supplement: S9 Fig — (DOCX) [file pone.0294683.s011.docx]

**
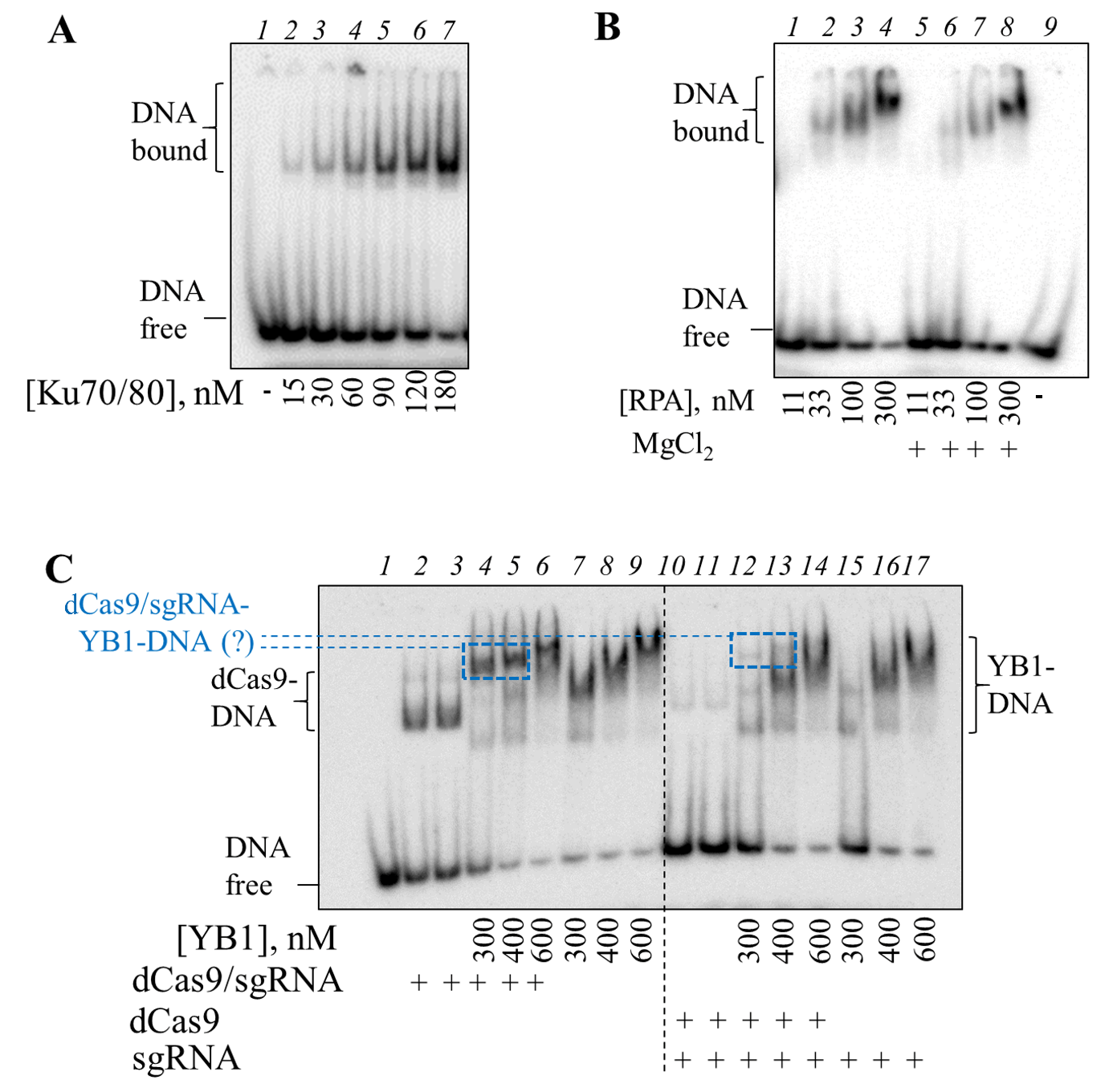
S9 Fig. Binding of Ku70/80, RPA and YB1 to the dsDNA substrate of Cas9.** The reaction mixtures containing 10 nM dsDNA 1/2* and Ku70/80 (A) or RPA (B) at increasing concentrations were incubated at 4°C for 30 min and separated in a native 5% PAG. Binding of RPA was explored in the absence or presence of 10 mM MgCl_2_. The K_d_ values for RPA were 90 ± 20 nM (without Mg^2+^) and 125 ± 40 nM (with Mg^2+^), respectively. The DNA binding activity of YB1 (C) was explored in the absence (lanes 7–9) and presence of dCas9 (20 nM) and sgRNA (20 nM), added separately or together.
