# Supplementary material for: Cas9 is mostly orthogonal to human systems of DNA break sensing and repair
Source: PLoS One. 2023 Nov 29;18(11):e0294683. doi: 10.1371/journal.pone.0294683 (PMC10686484; doi:10.1371/journal.pone.0294683)
Supplement: S10 Fig — (DOCX) [file pone.0294683.s012.docx]

**S10 Fig. Cleavage of the pMSH2 plasmid substrate by Cas9/sgRNA_pMSH2_ in the presence of different proteins.** The DNA plasmid substrate corresponding to sgRNA_pMSH2_ was constructed based on a plasmid with inserted *hMSH2* gene (Addgene ID #16453). The sequence of sgRNA_pMSH2_ is presented below:

5′‑pppAUCAAGUACAUGGGGCCGGCGUUUUAGAGCUAGAAAUAGCAAGUUAAAAUAAGGCUAGUCCGUUAUCAACUUGAAAAAGUGGCACCGAGUCGGUGCUUUU-3′. A) Cleavage of two different DNA plasmid substrates by Cas9/sgRNA. Cas9/sgRNA_pLK1_ (20 nM) or Cas9/sgRNA_pMSH2_ (20 nM) was incubated with pLK1 DNA (10 ng/µl) or pMSH2 DNA (10 ng/µl), respectively. S, substrate (supercoiled plasmid pMSH2 or pLK1); P1, SSB containing product (nicked plasmids); P2, DSB-containing product (linear plasmids). The curves show the accumulation of P1+P2 products under the indicated conditions. The sizes of DNA markers are shown next to the gel images.
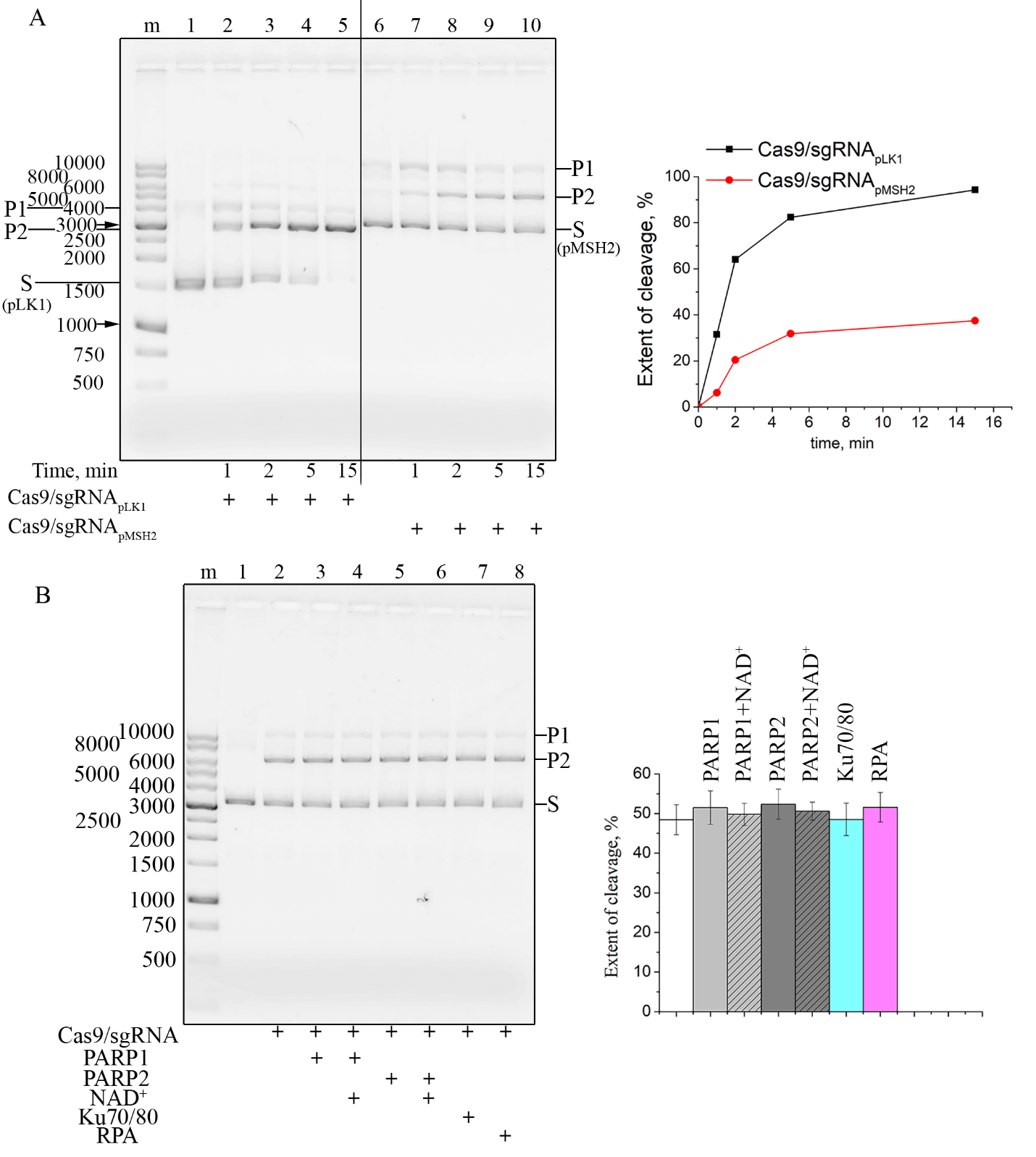
 B) Cas9/sgRNA_pMSH2_ (20 nM) was incubated with pMSH2 DNA (10 ng/µl) in the absence and presence of PARP1 (100 nM) or PARP2 (500 nM) without or with 500 μM NAD^+^, Ku70/80 (250 nM), and RPA (500 nM) for 15 min at 37°C. S, substrate (supercoiled pMSH2 plasmid); P1, SSB containing product (nicked plasmid); P2, DSB-containing product (linear plasmid). The bar chart shows the extent of pMSH2 DNA cleavage in the presence of the indicated proteins.
