# Supplementary material for: Cas9 is mostly orthogonal to human systems of DNA break sensing and repair
Source: PLoS One. 2023 Nov 29;18(11):e0294683. doi: 10.1371/journal.pone.0294683 (PMC10686484; doi:10.1371/journal.pone.0294683)
Supplement: S11 Fig — (DOCX) [file pone.0294683.s013.docx]

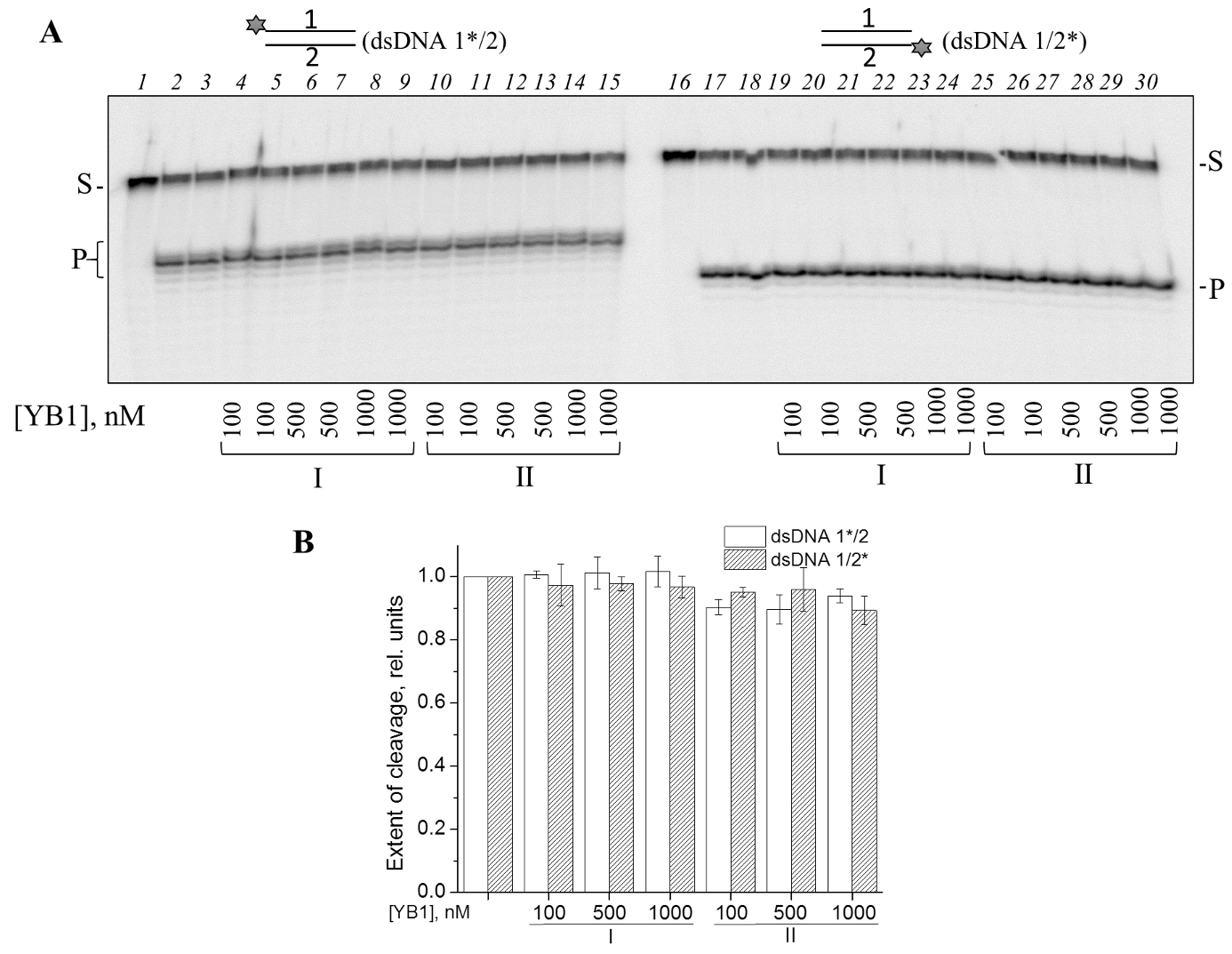


**S11 Fig. Effects of YB1 on the cleavage activity of Cas9**. (A) The endonuclease activity was tested by incubation of Cas9/sgRNA (20 nM) with dsDNA 1*/2 or dsDNA 1/2* (10 nM) at 37°C for 30 min, in the absence (lanes 2, 3 and 17, 18) and presence (lanes 4–15 and 19–30) of YB1 (100-1000 nM). In the presence of YB1, the reaction was performed without pre-incubation (I) or with pre-incubation of Cas9/sgRNA with YB1 on ice for 30 min and following addition of dsDNA 1/2 (II). The reaction products were separated in a denaturing 20% PAG. (B) Bar charts show the relative extent of dsDNA 1/2 cleavage induced by Cas9 in the specified reaction conditions (normalized to that in the control sample containing no YB1; the mean ± SD, *n* = 3).
