# Supplementary material for: Cas9 is mostly orthogonal to human systems of DNA break sensing and repair
Source: PLoS One. 2023 Nov 29;18(11):e0294683. doi: 10.1371/journal.pone.0294683 (PMC10686484; doi:10.1371/journal.pone.0294683)

Fig 1A.

Numbering of the lanes corresponds to that in Fig 1A.

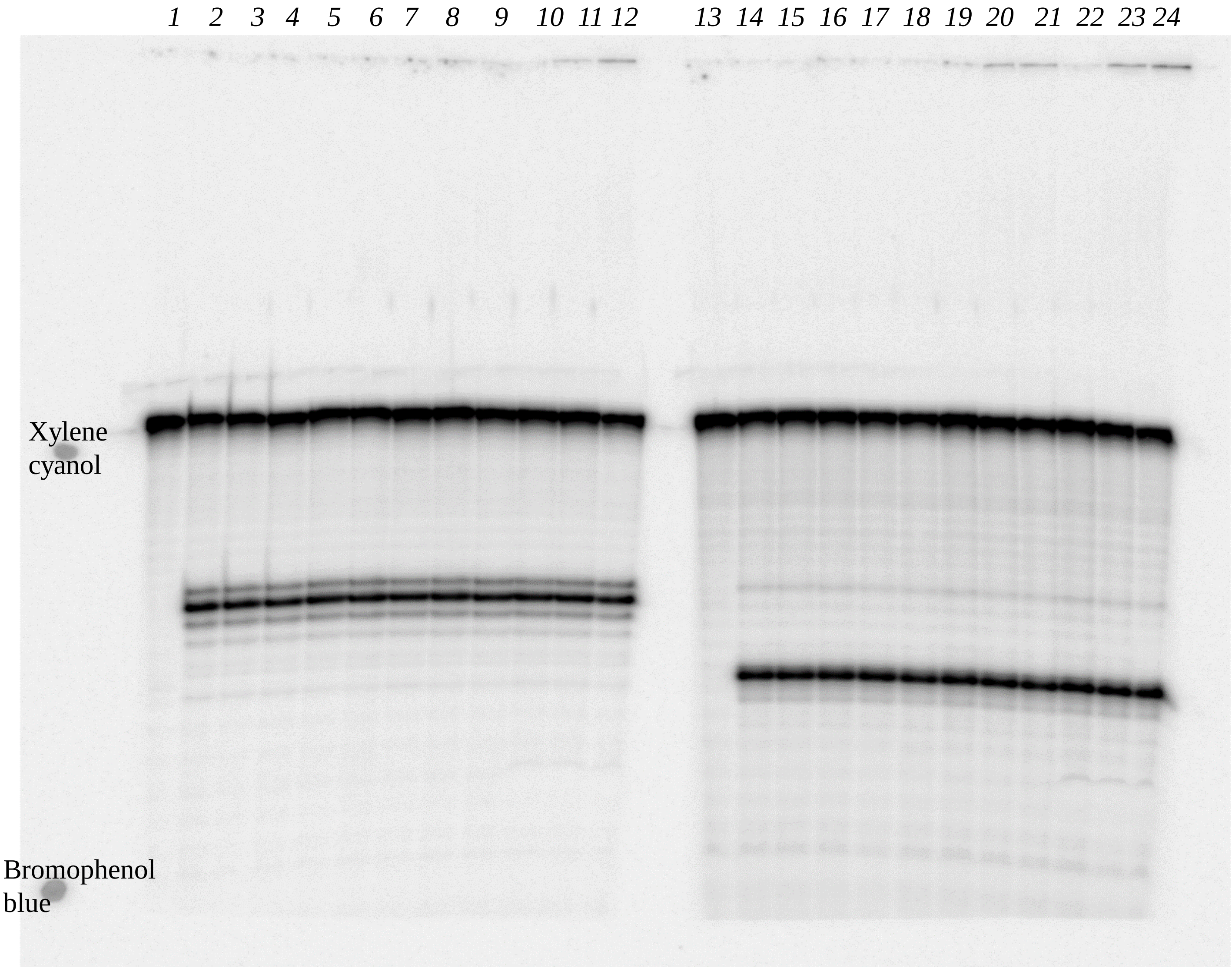

Fig 1B.
Numbering of the lanes corresponds to that in Fig 1B.

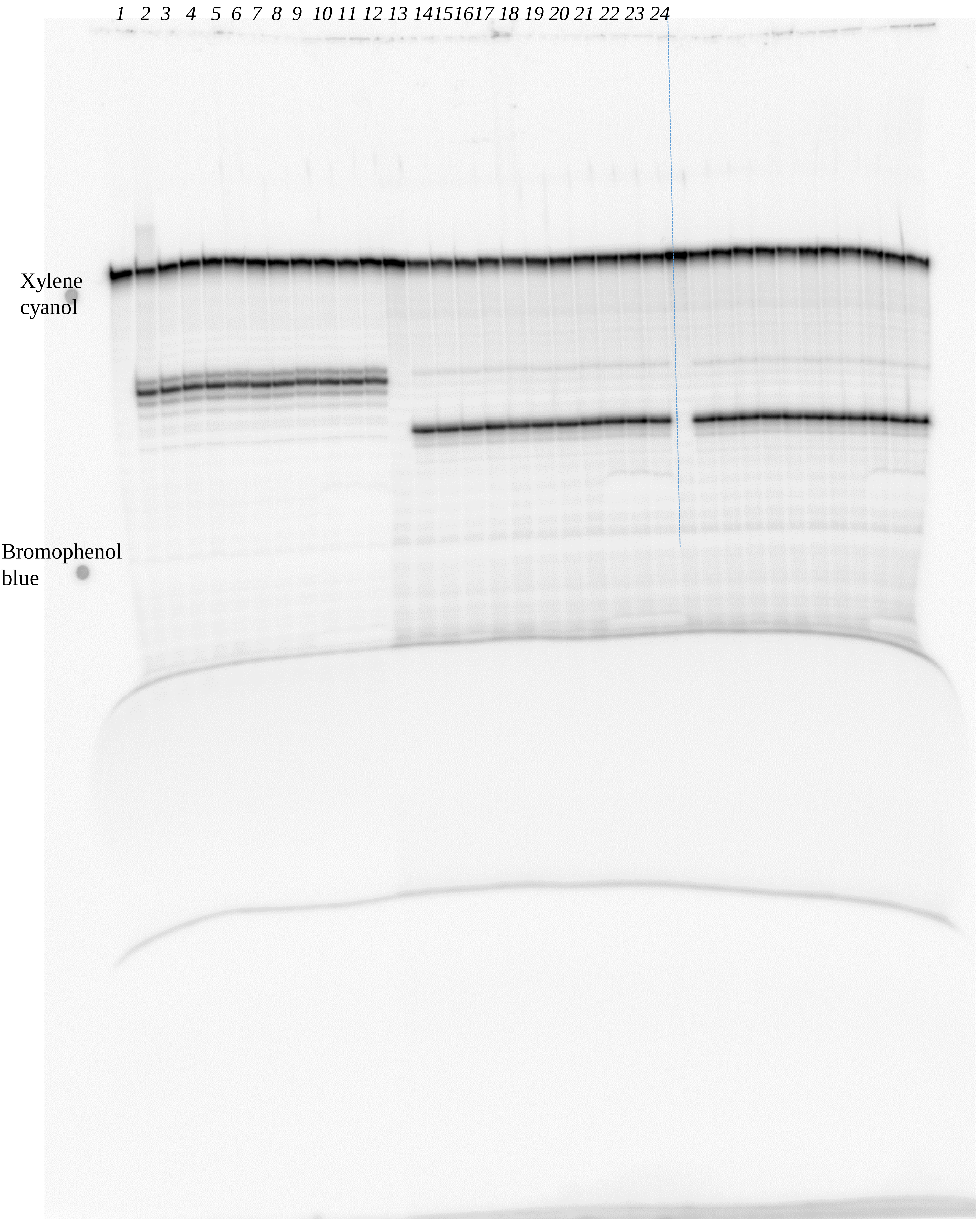

Fig 1C

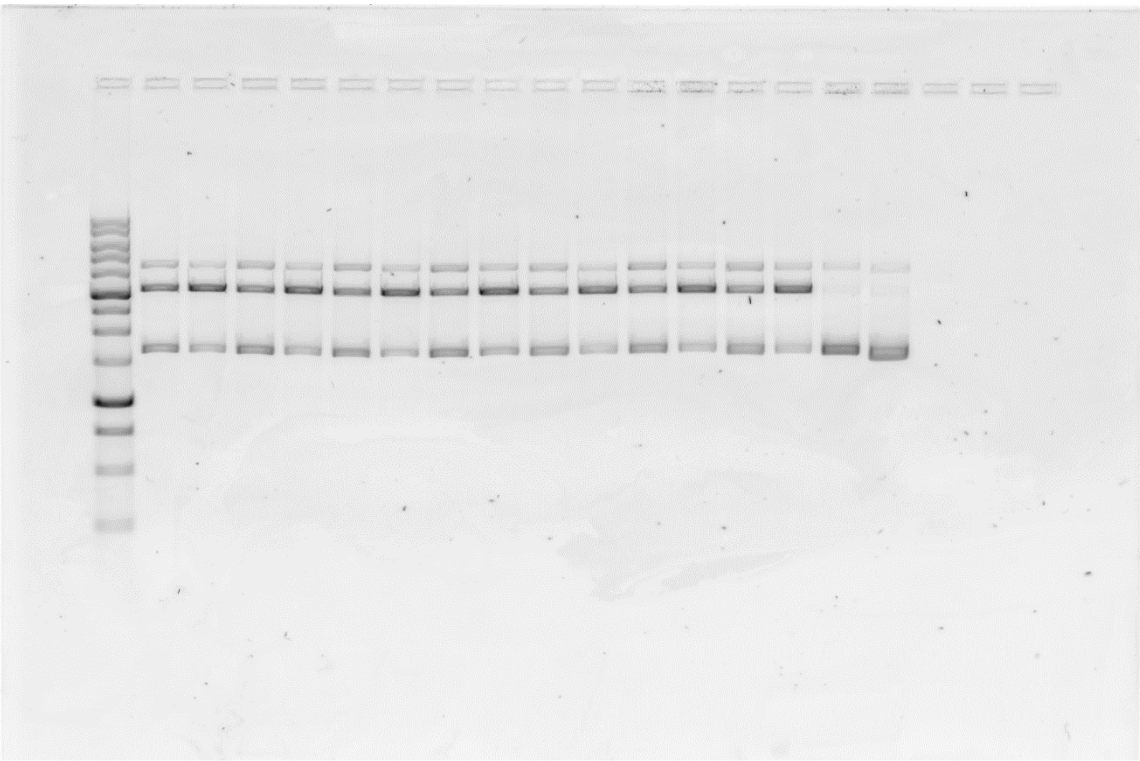

Fig 1D

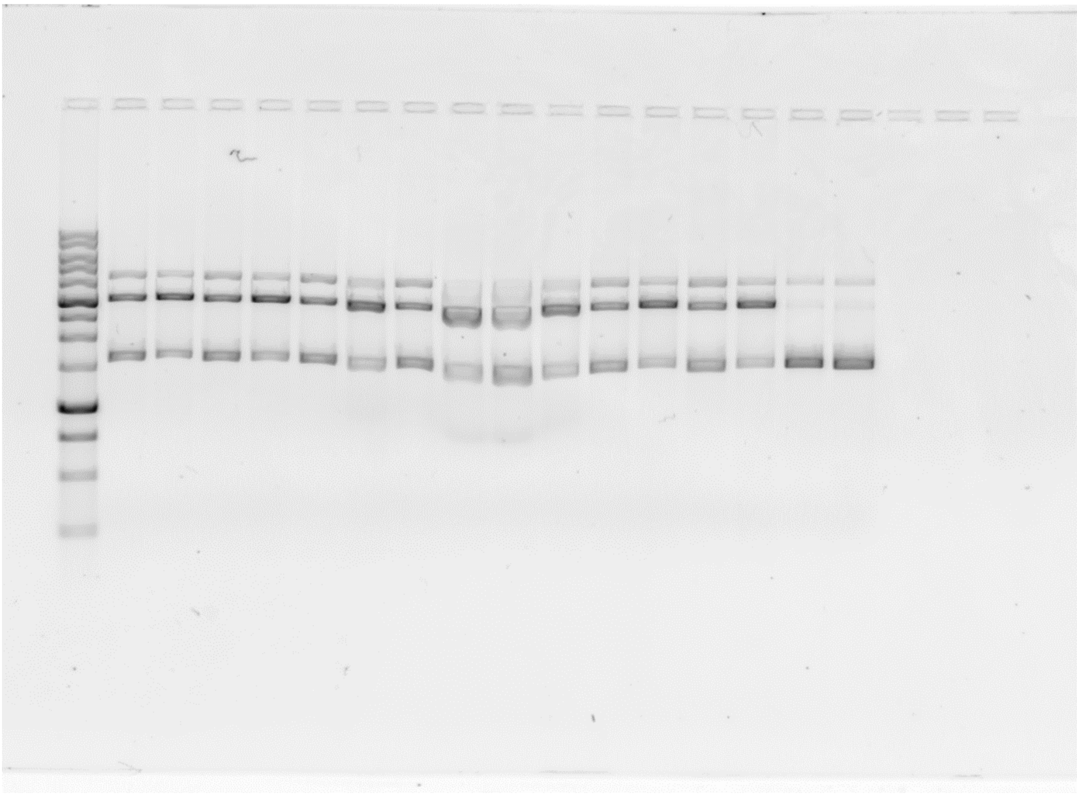

Figs 2A and 2B.
Numbering of the lanes corresponds to that in Figs 2A and 2B.

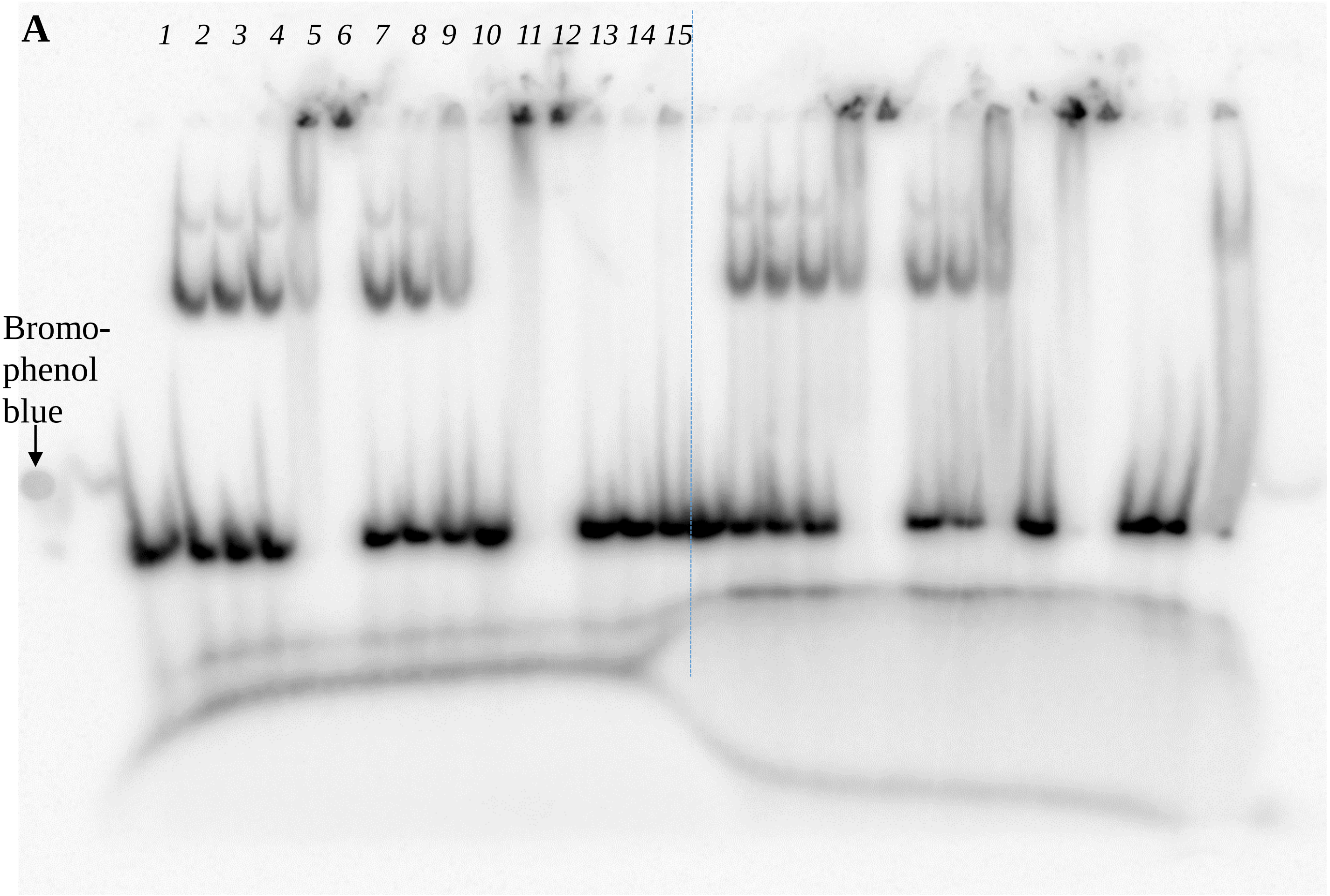

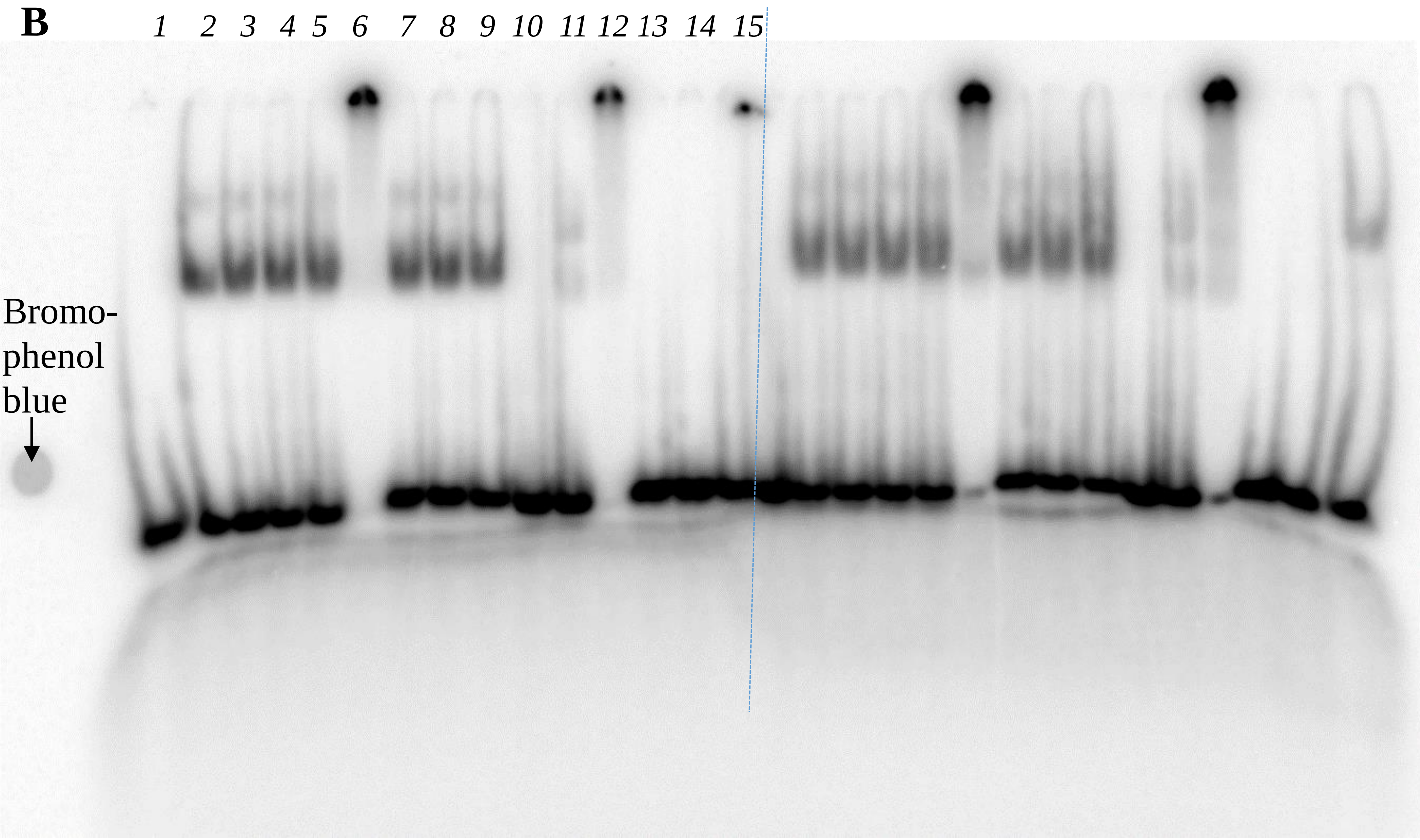

Fig 2C

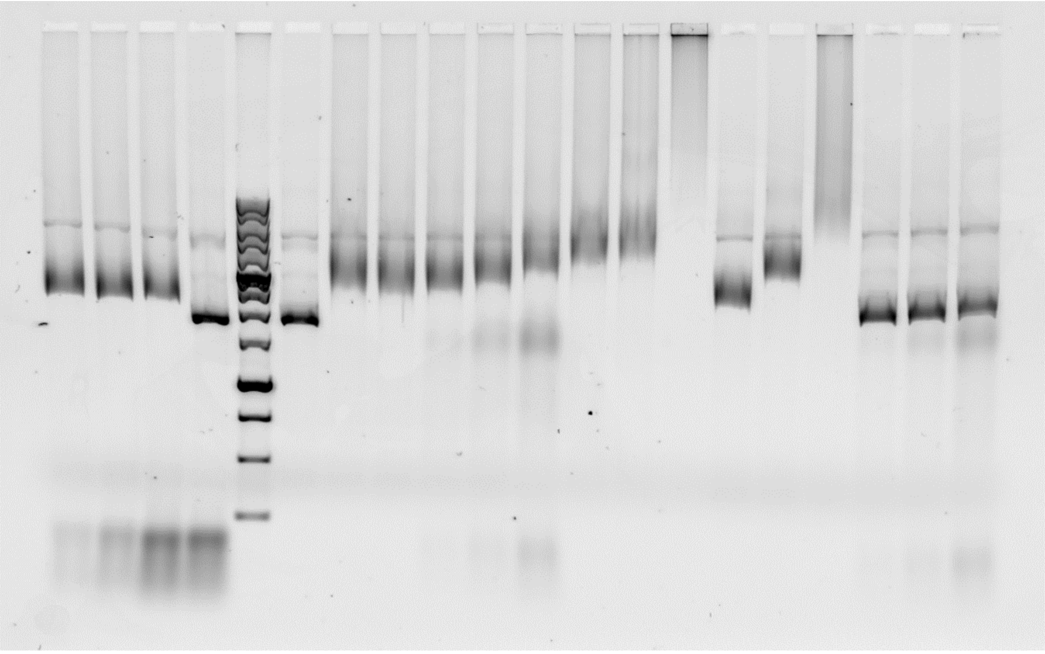

Fig 3A

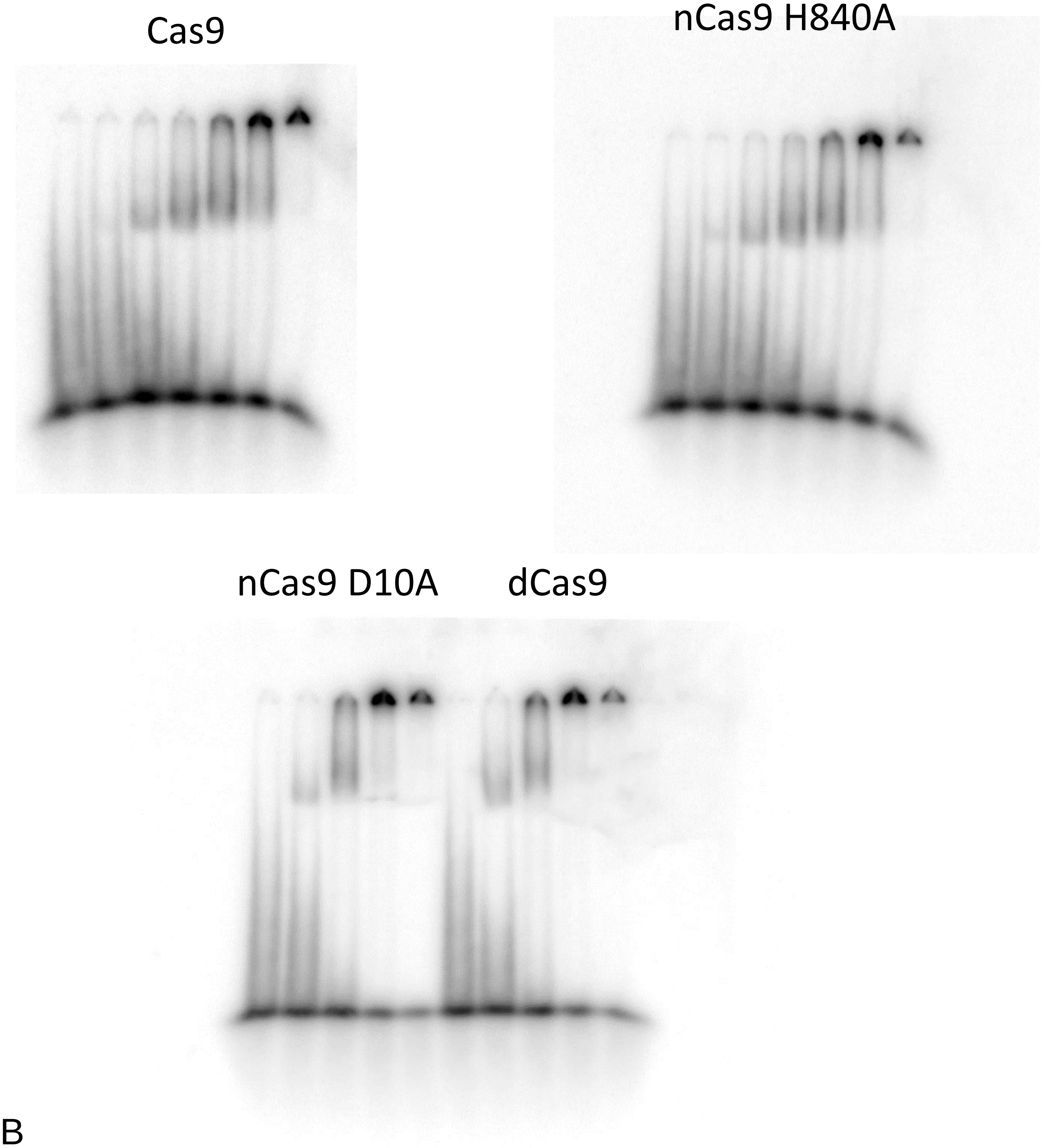

Fig 3B

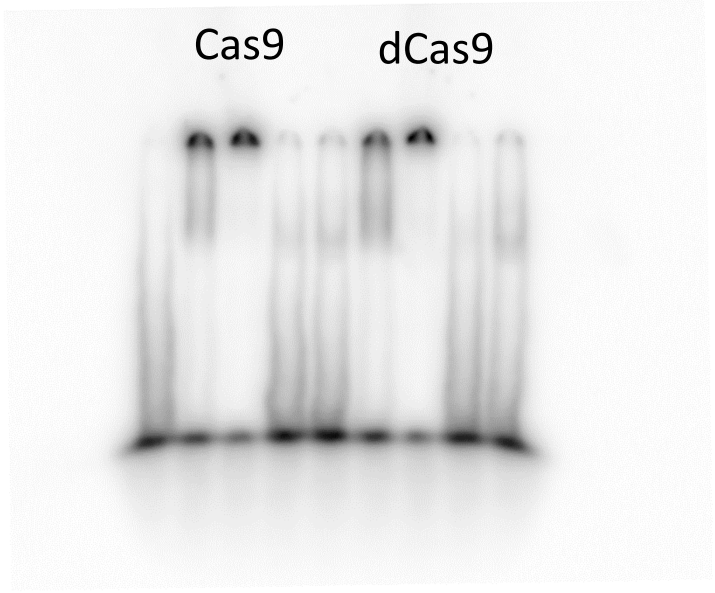

Fig 4A

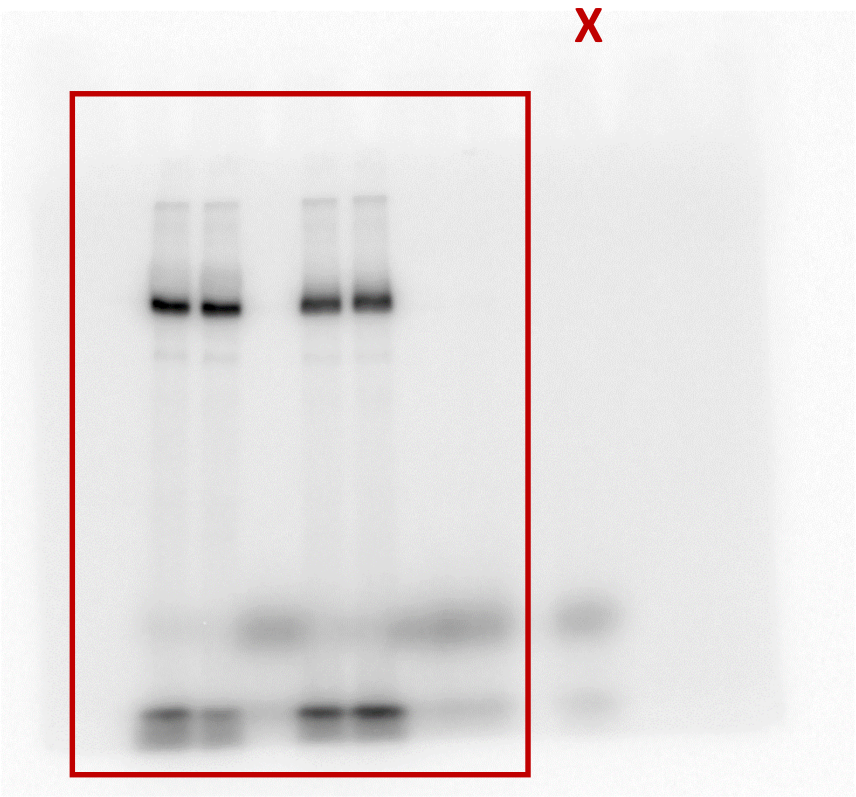

Fig 4B

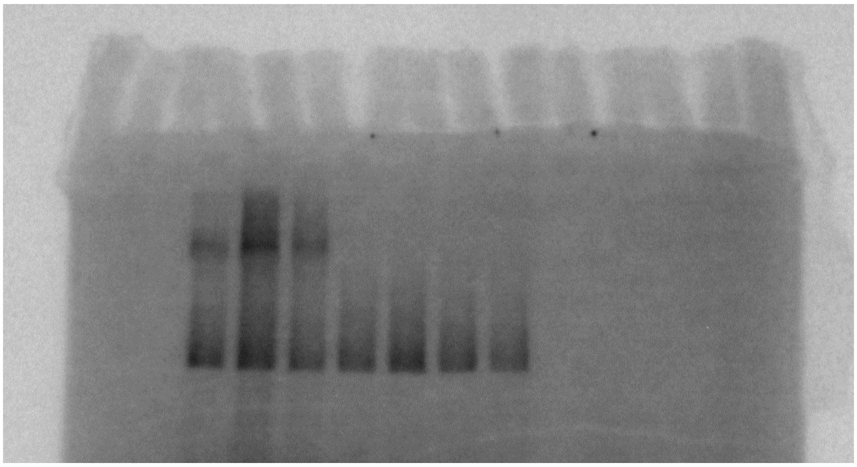

Long exposition (3 weeks)
of the same gel

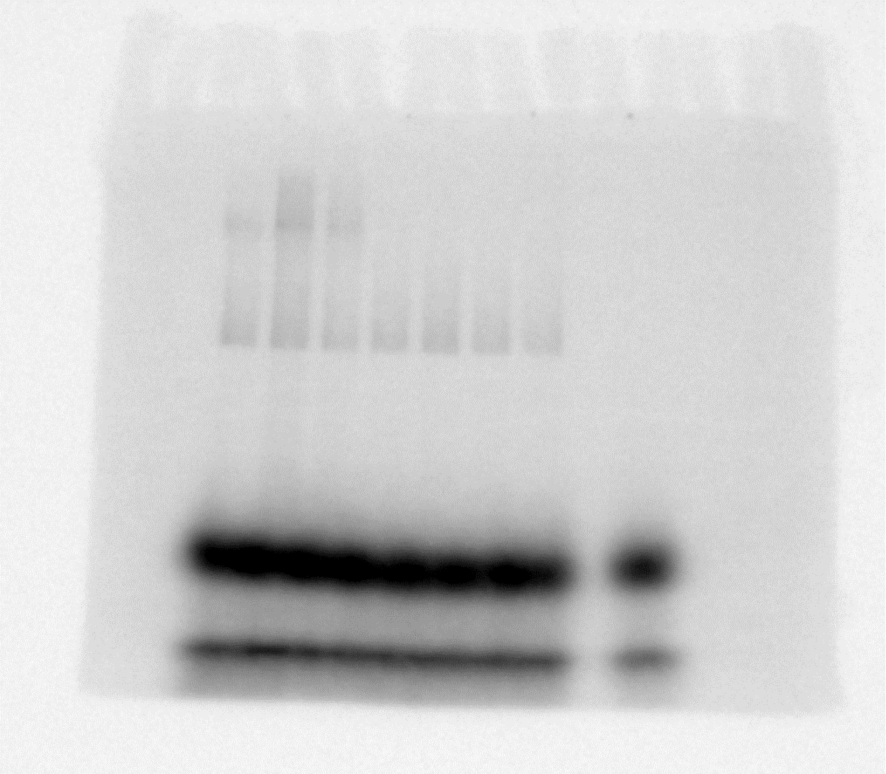

Fig 5

Long exposition (1 week) of the same gel

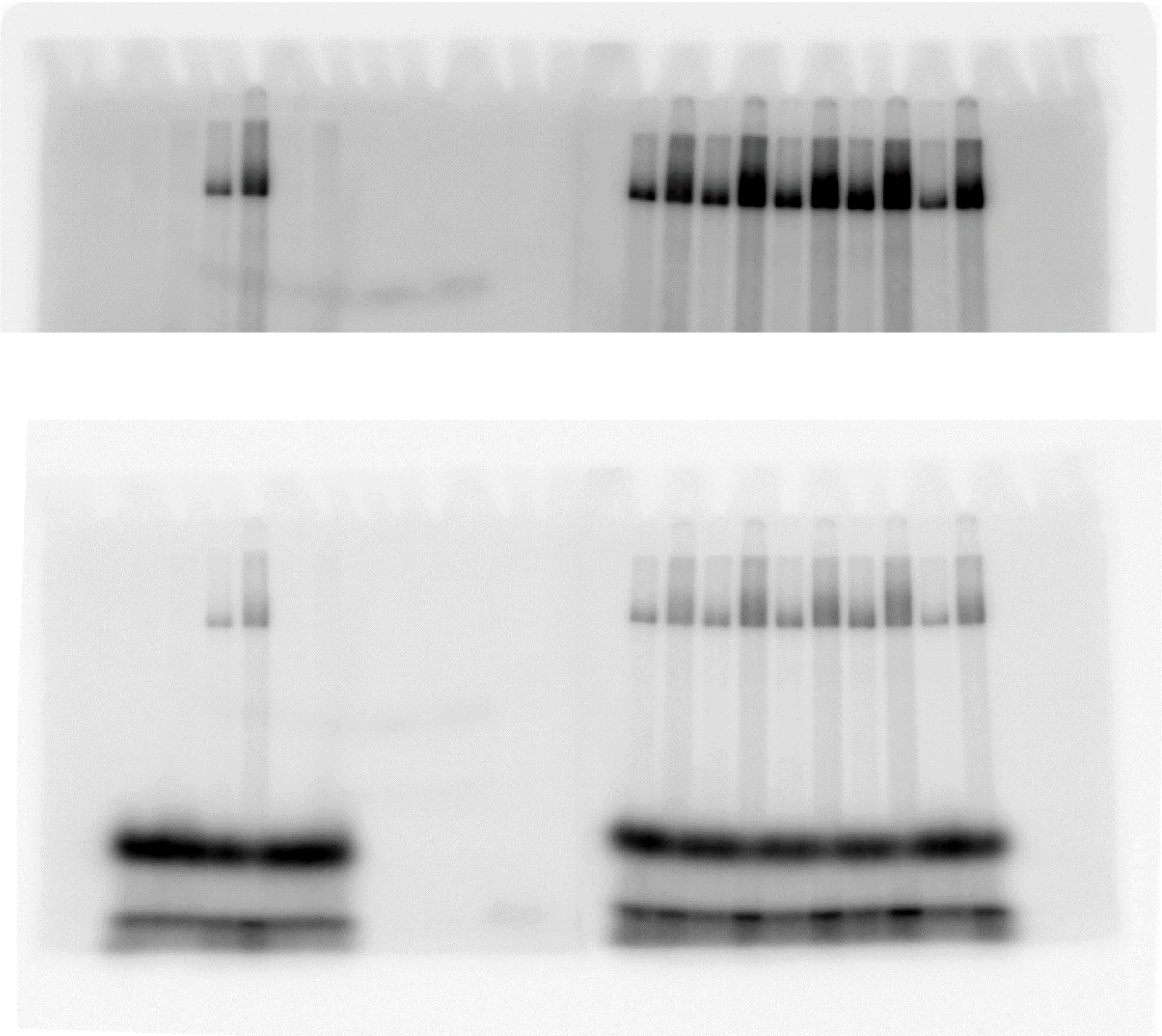

Fig 6A

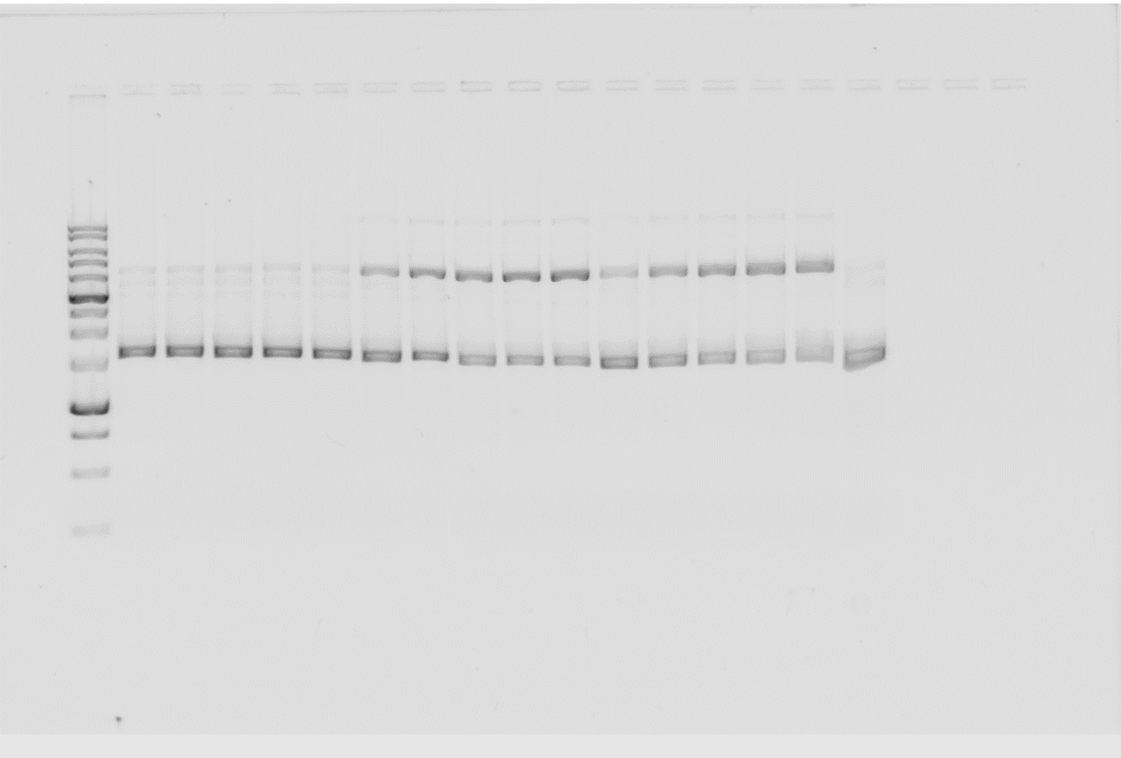

Fig 6B

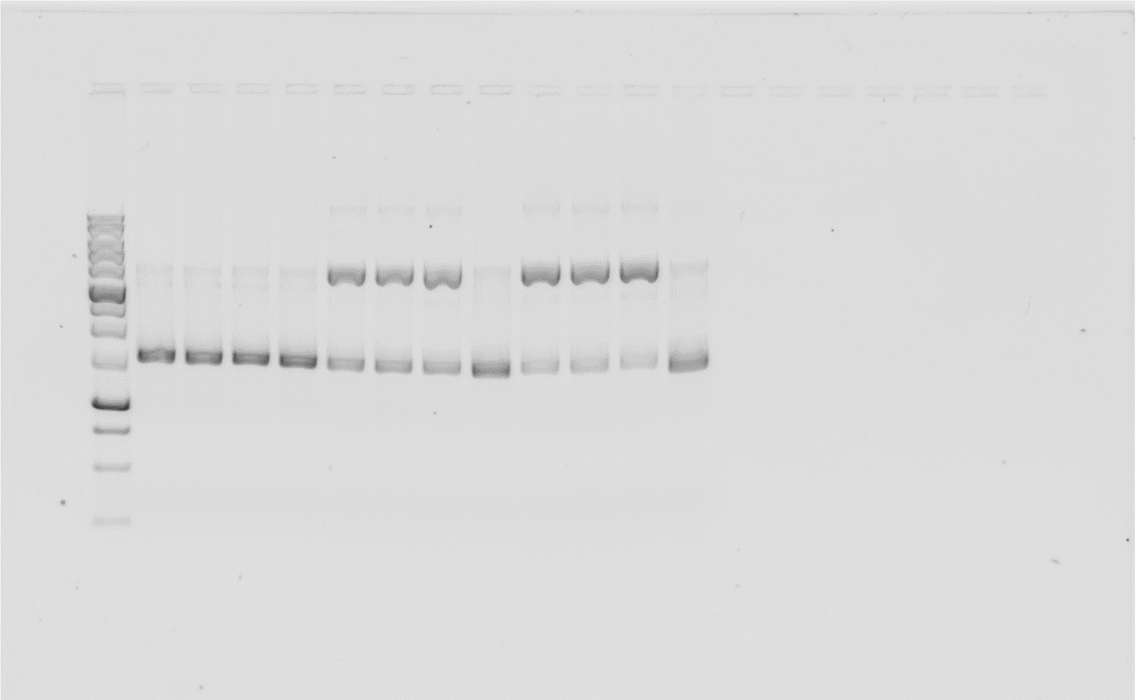

Fig 6C

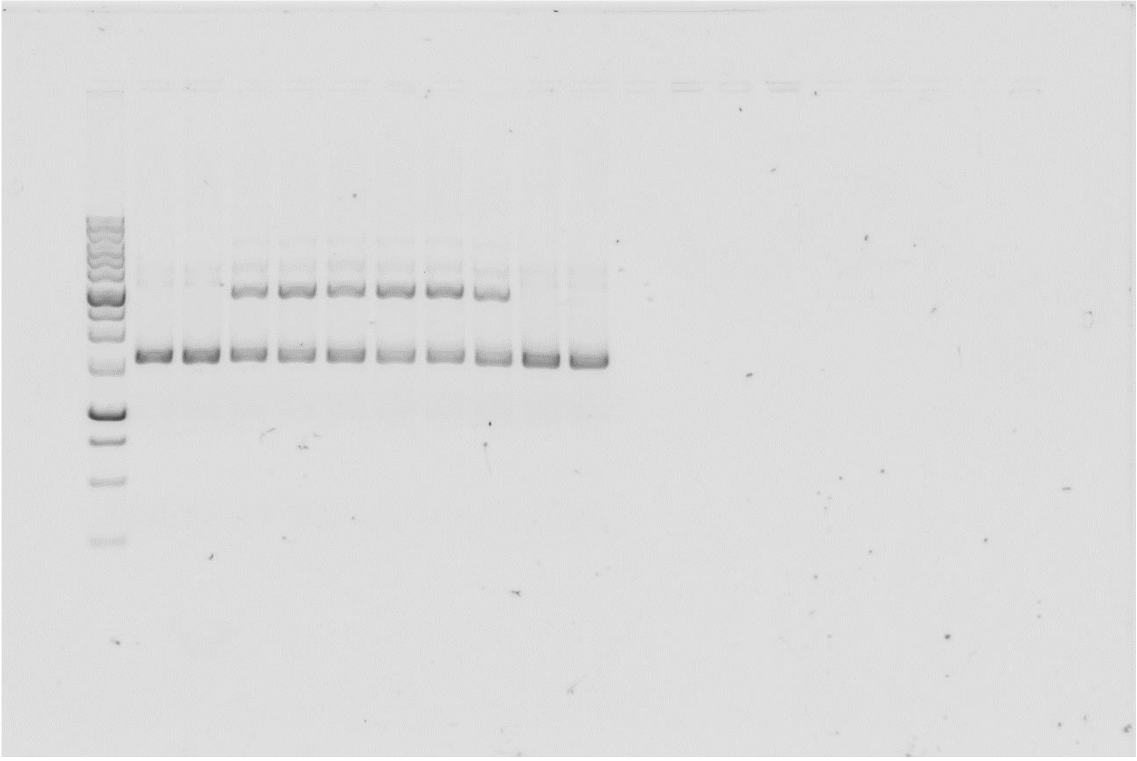

Fig 7A.
Numbering of the lanes corresponds to that in Fig 7A.

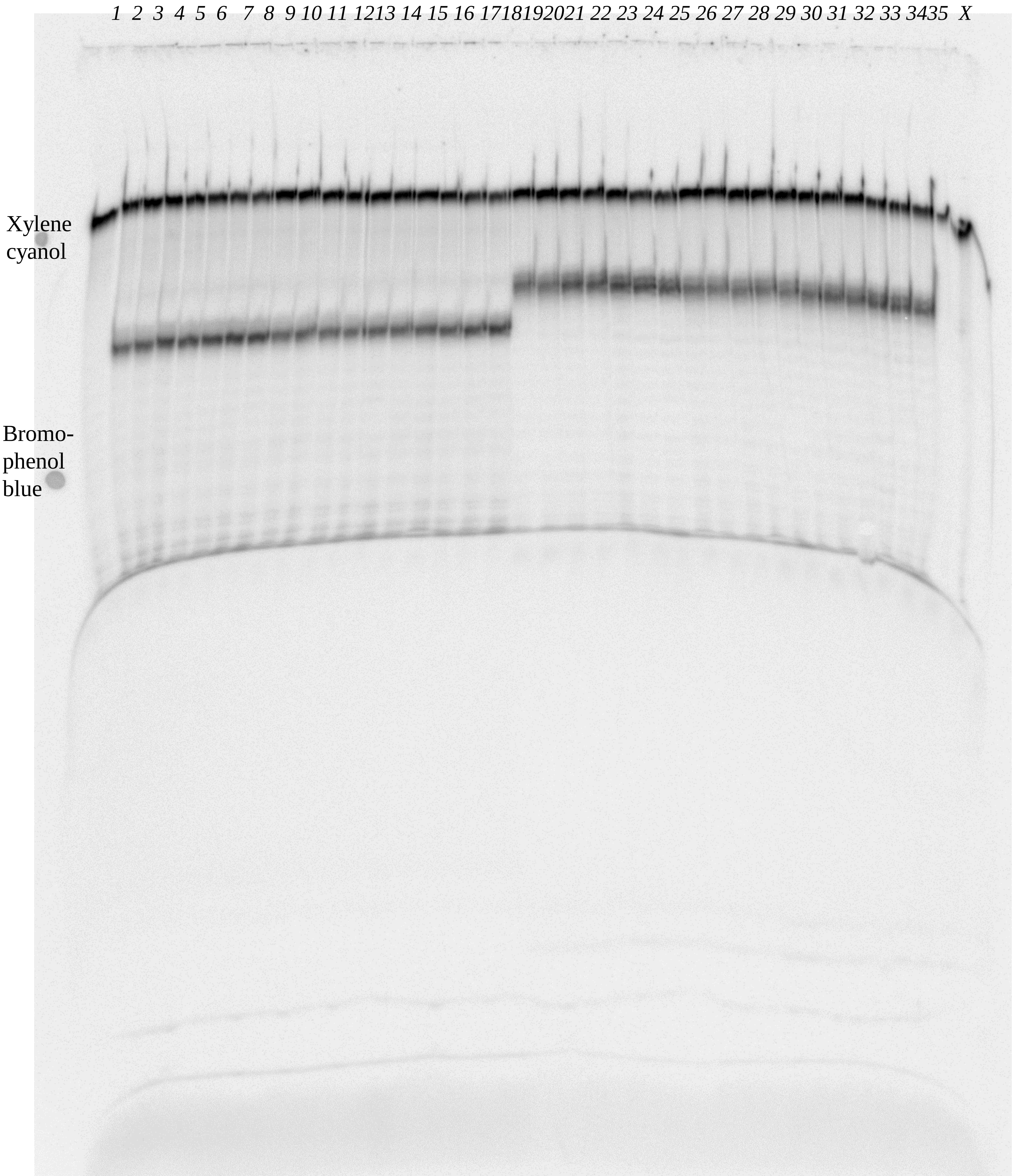

Fig 7C

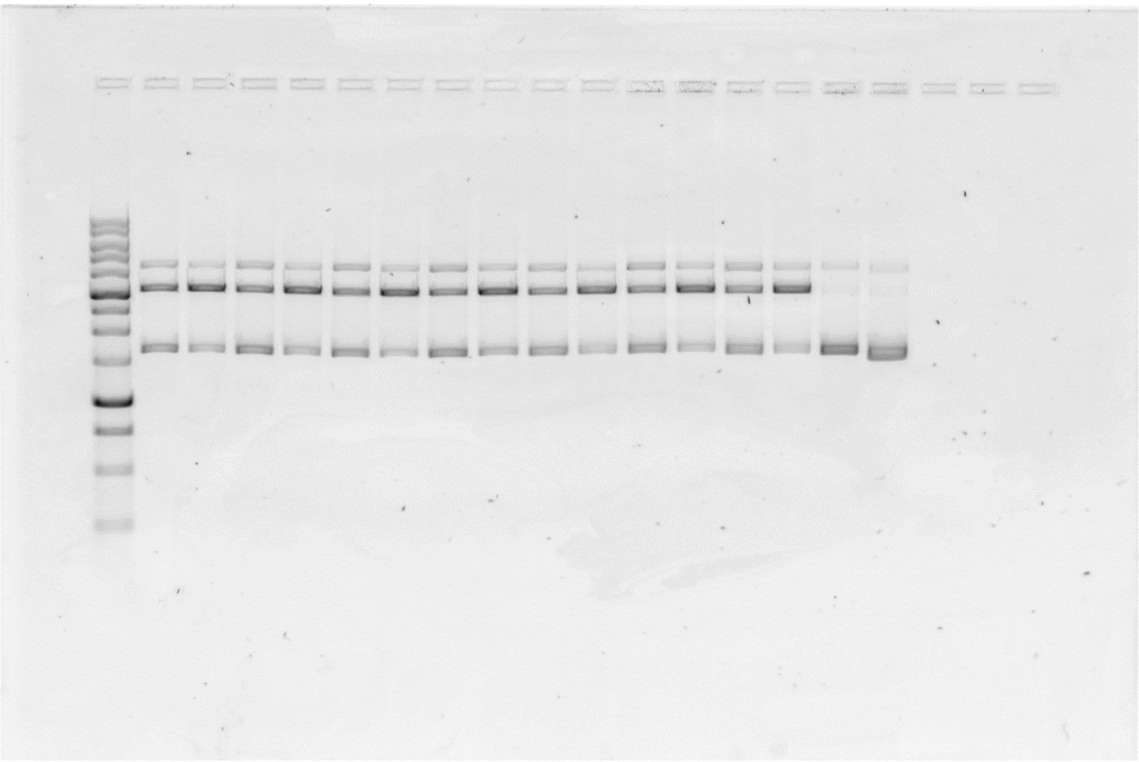

Fig 8A.
Numbering of the lanes corresponds to that in Fig 8A.

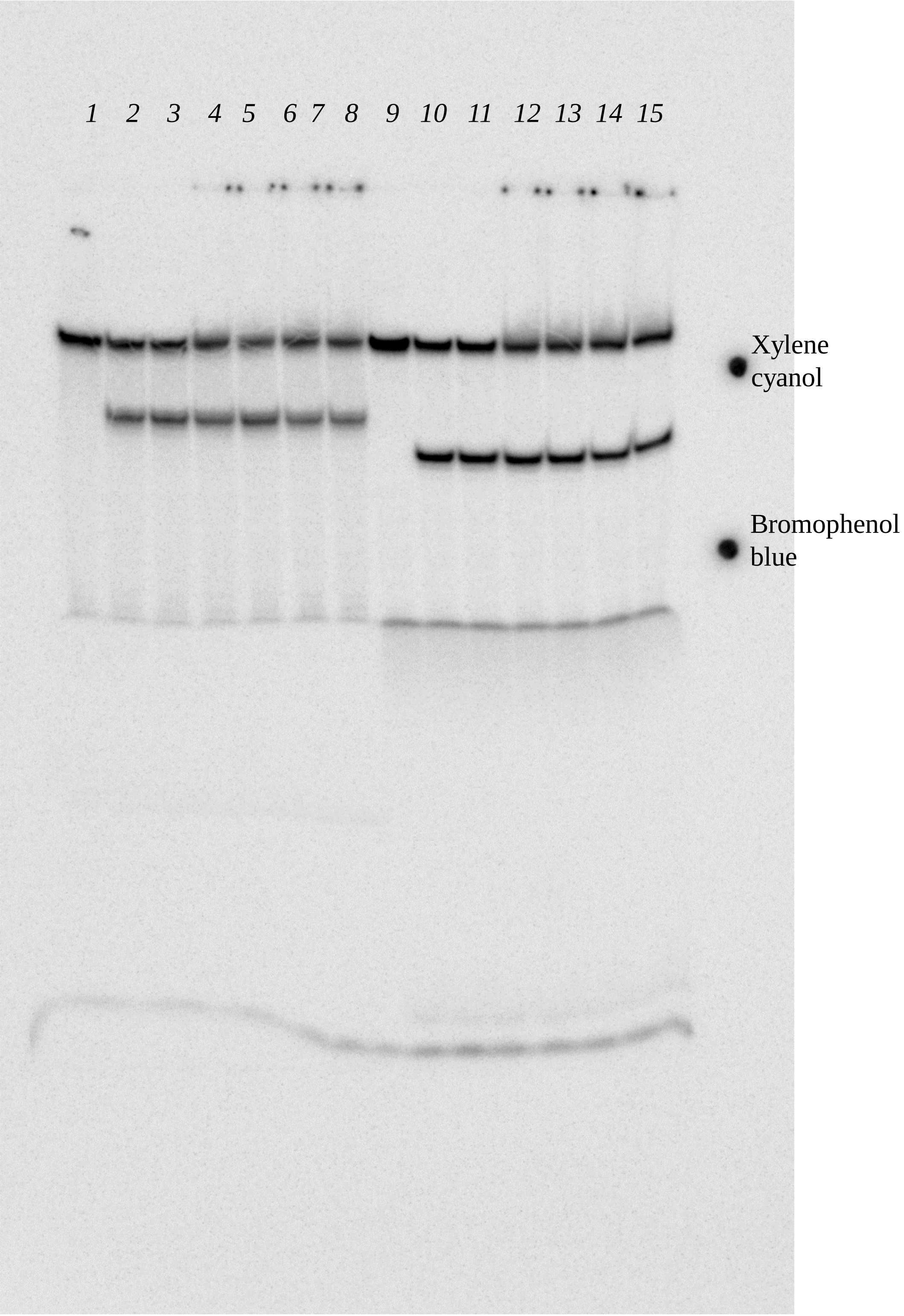

S2A Fig.

Numbering of the lanes corresponds to that in S2A Fig.

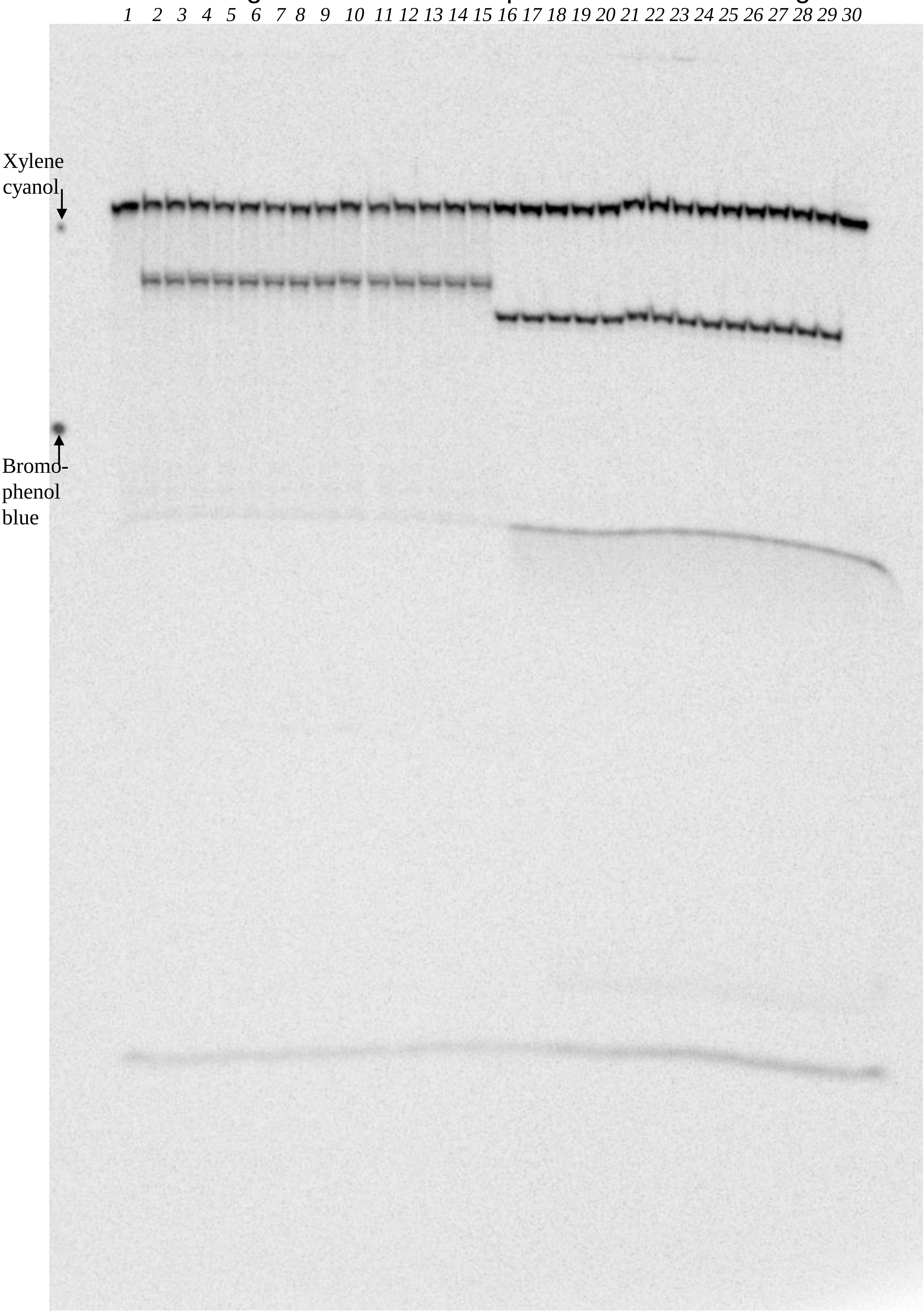

S3A Fig

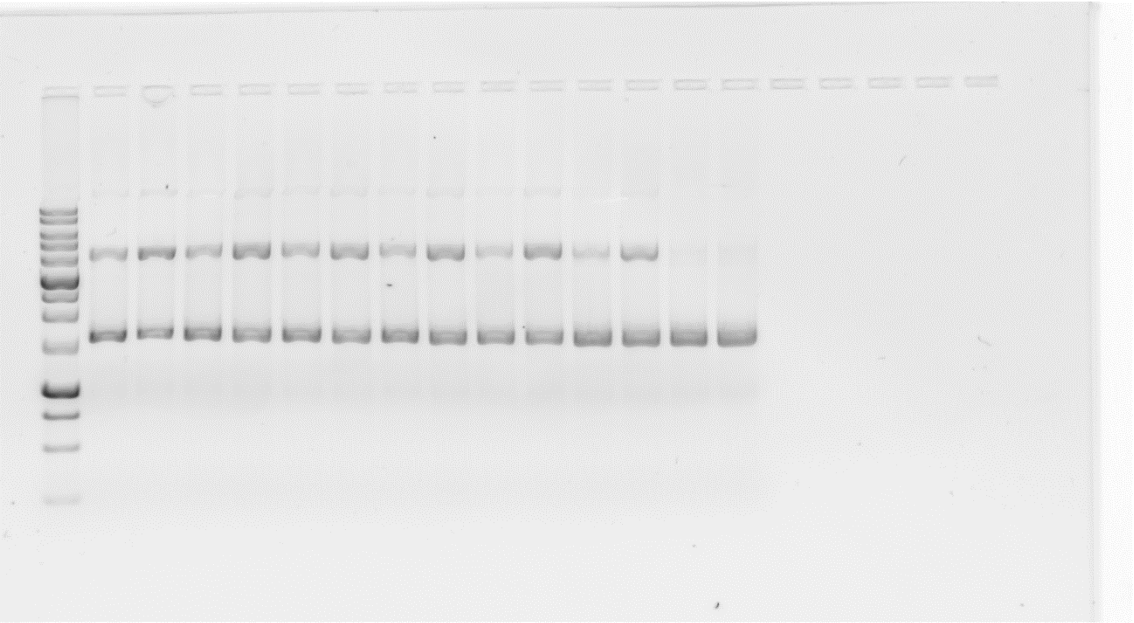

S3B Fig

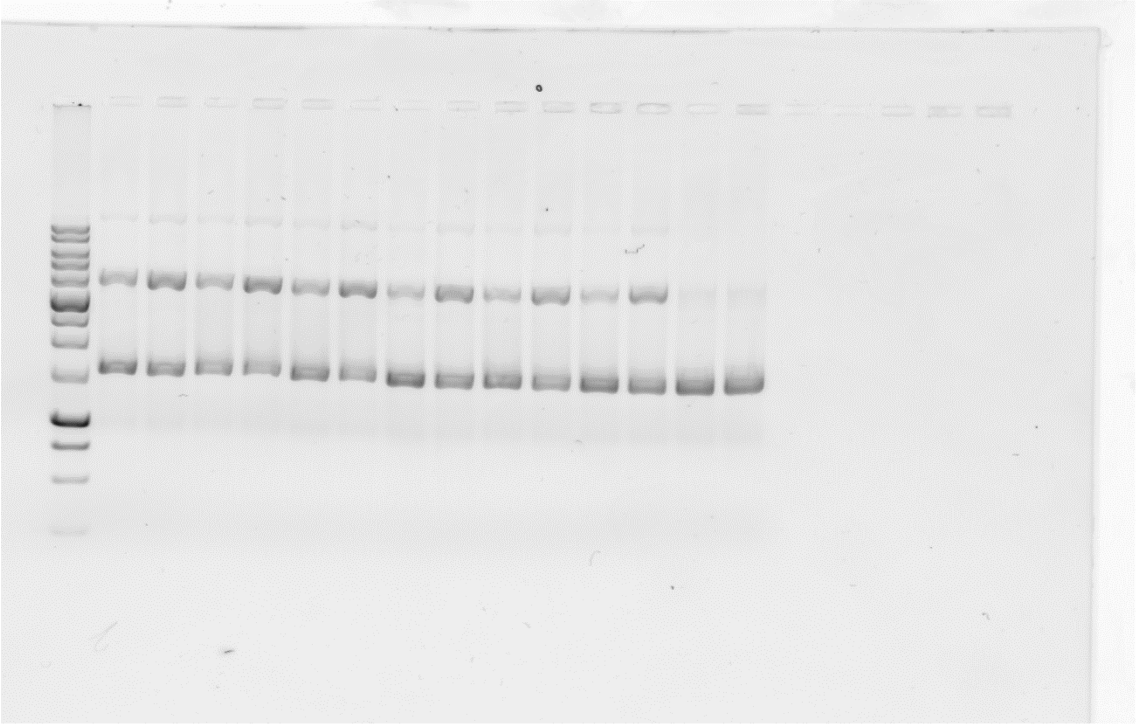

S4A Fig.

Numbering of the lanes corresponds to that in S4A Fig.

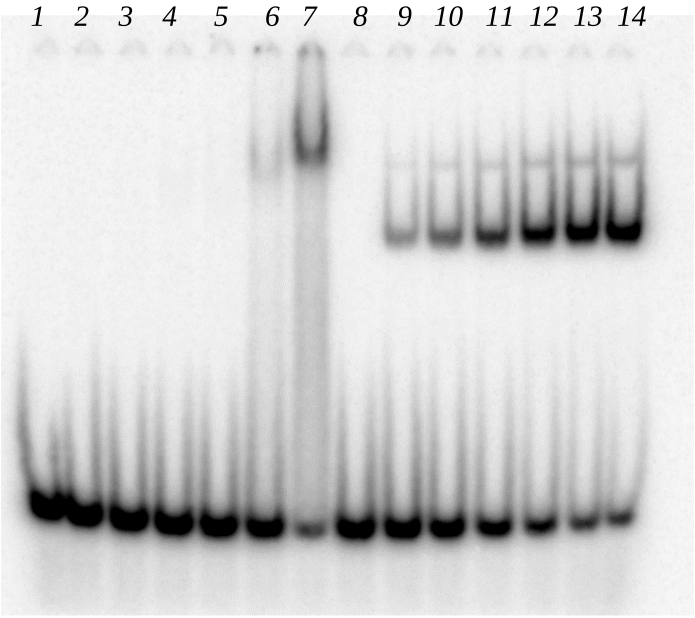

S4B and S4C Figs.
Numbering of the lanes corresponds to that in S4B and S4C Figs.

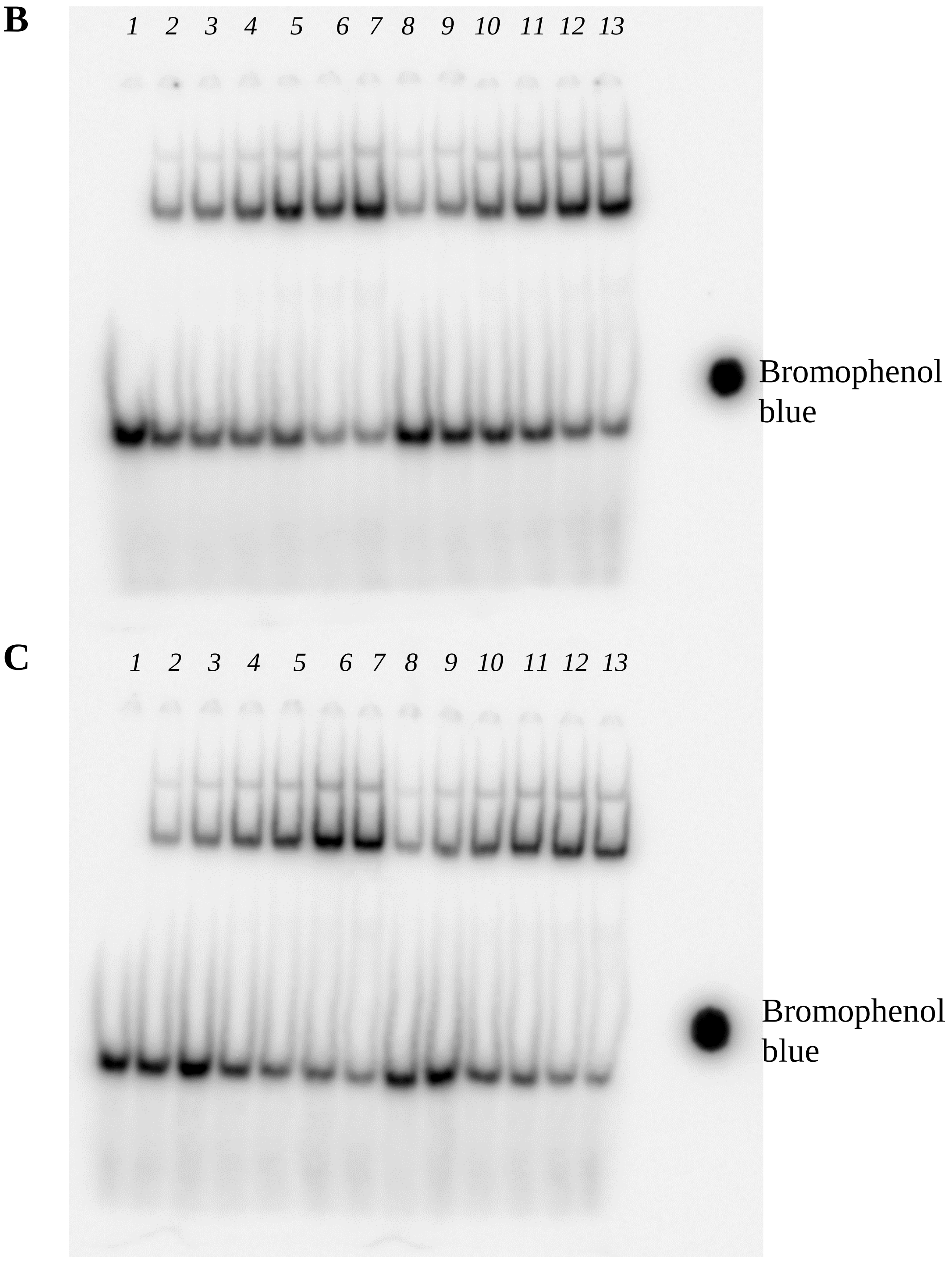

Fig S5A and Fig S5B.
Numbering of the lanes corresponds to that in Fig S5A and Fig S5B.

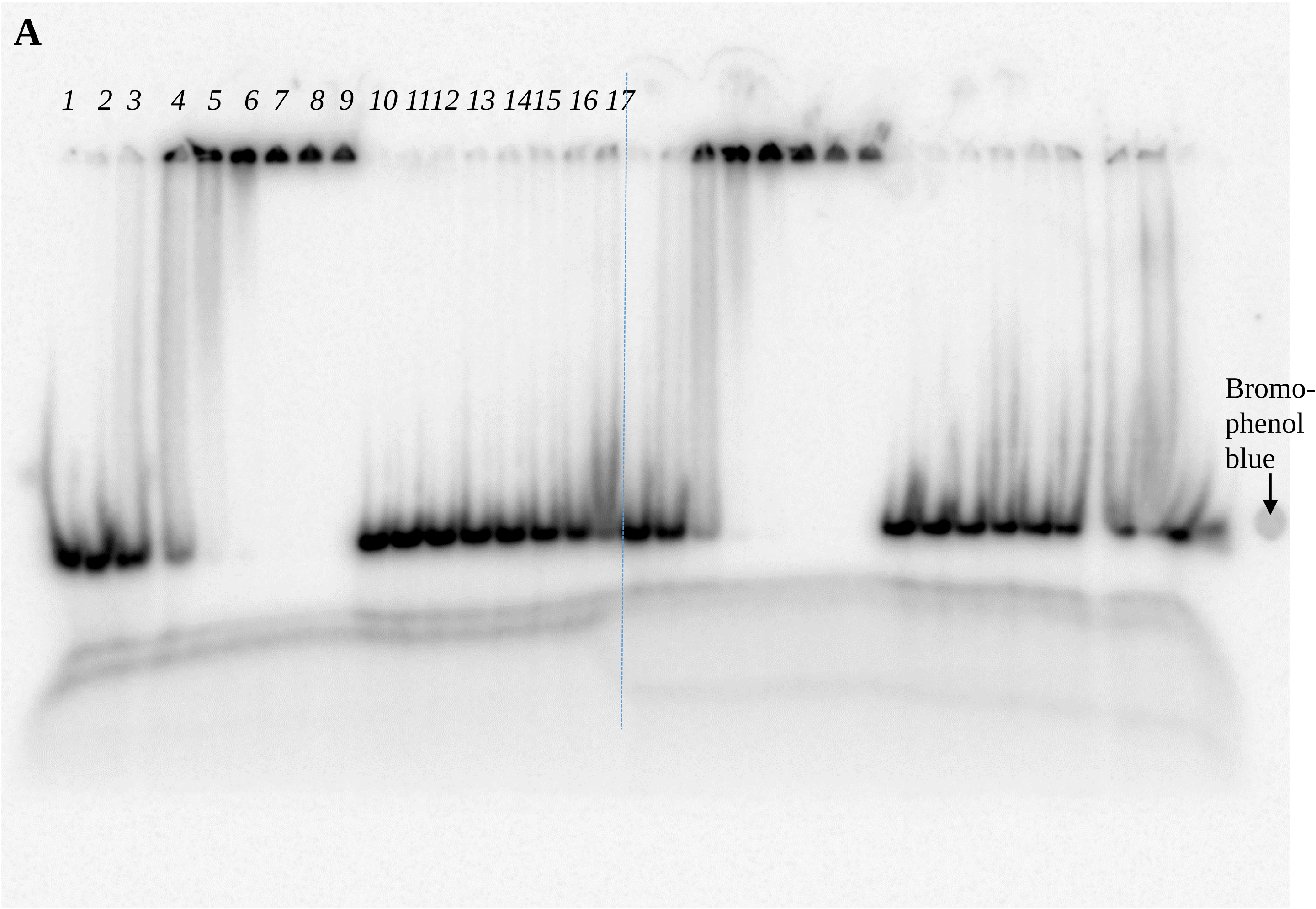

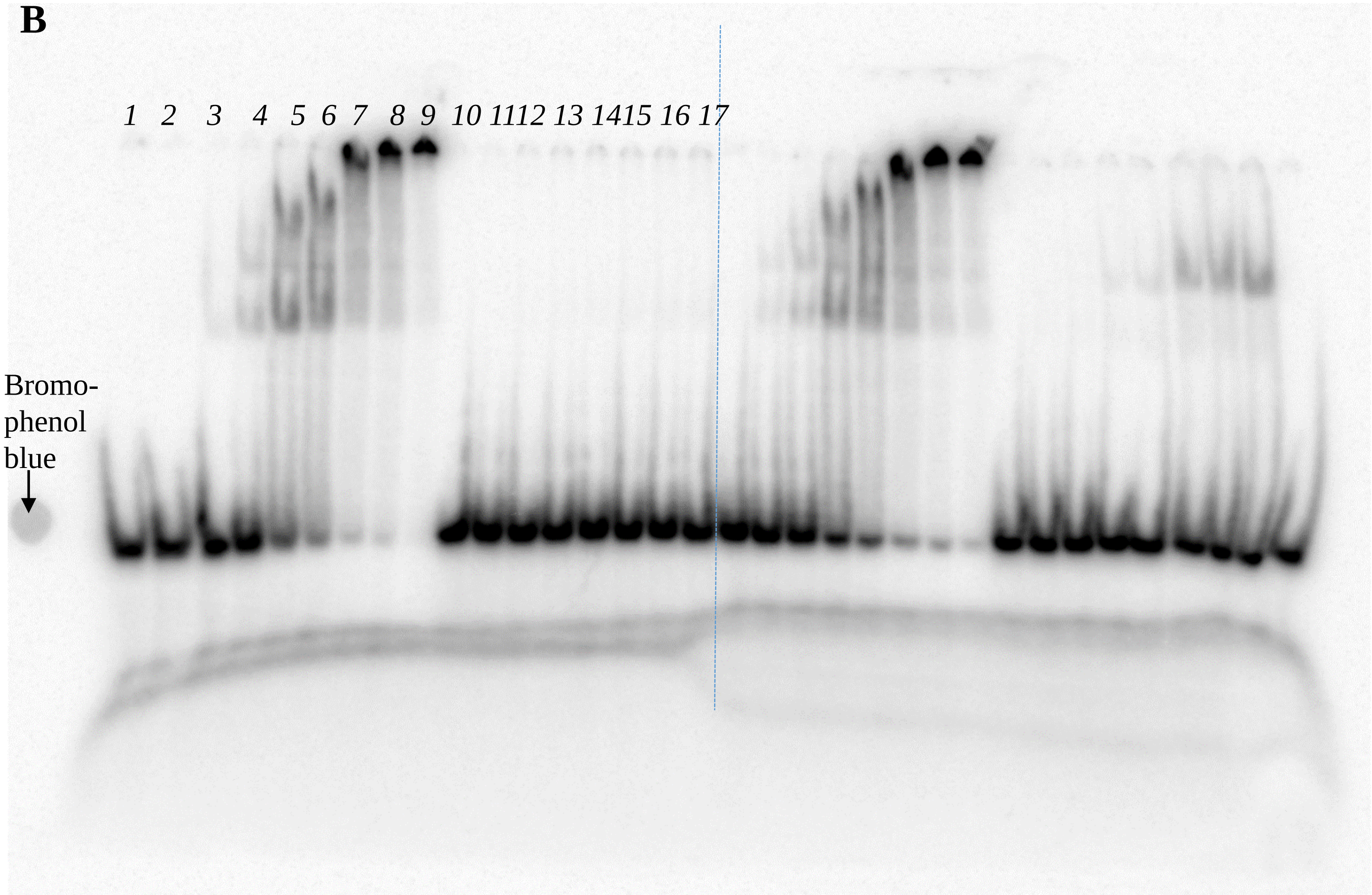

S6 Fig

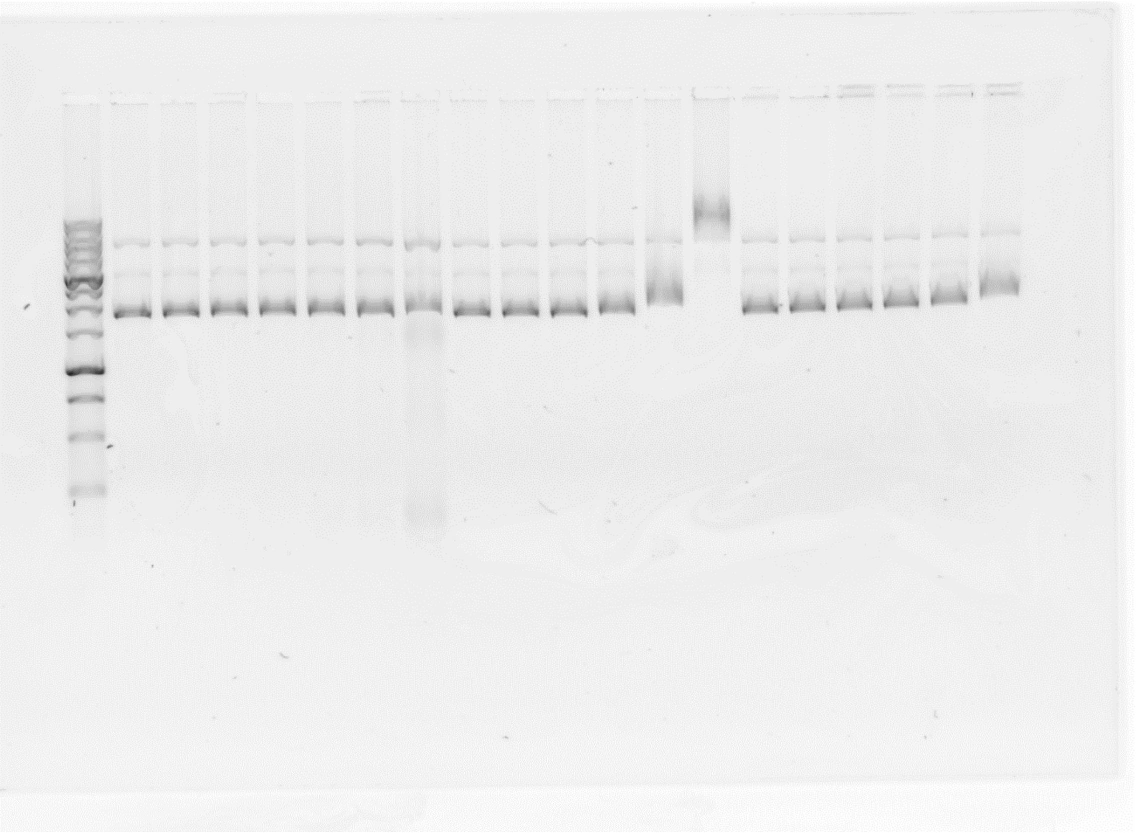

S7A Fig (top panel).

Numbering of the lanes corresponds to that in S7A Fig.

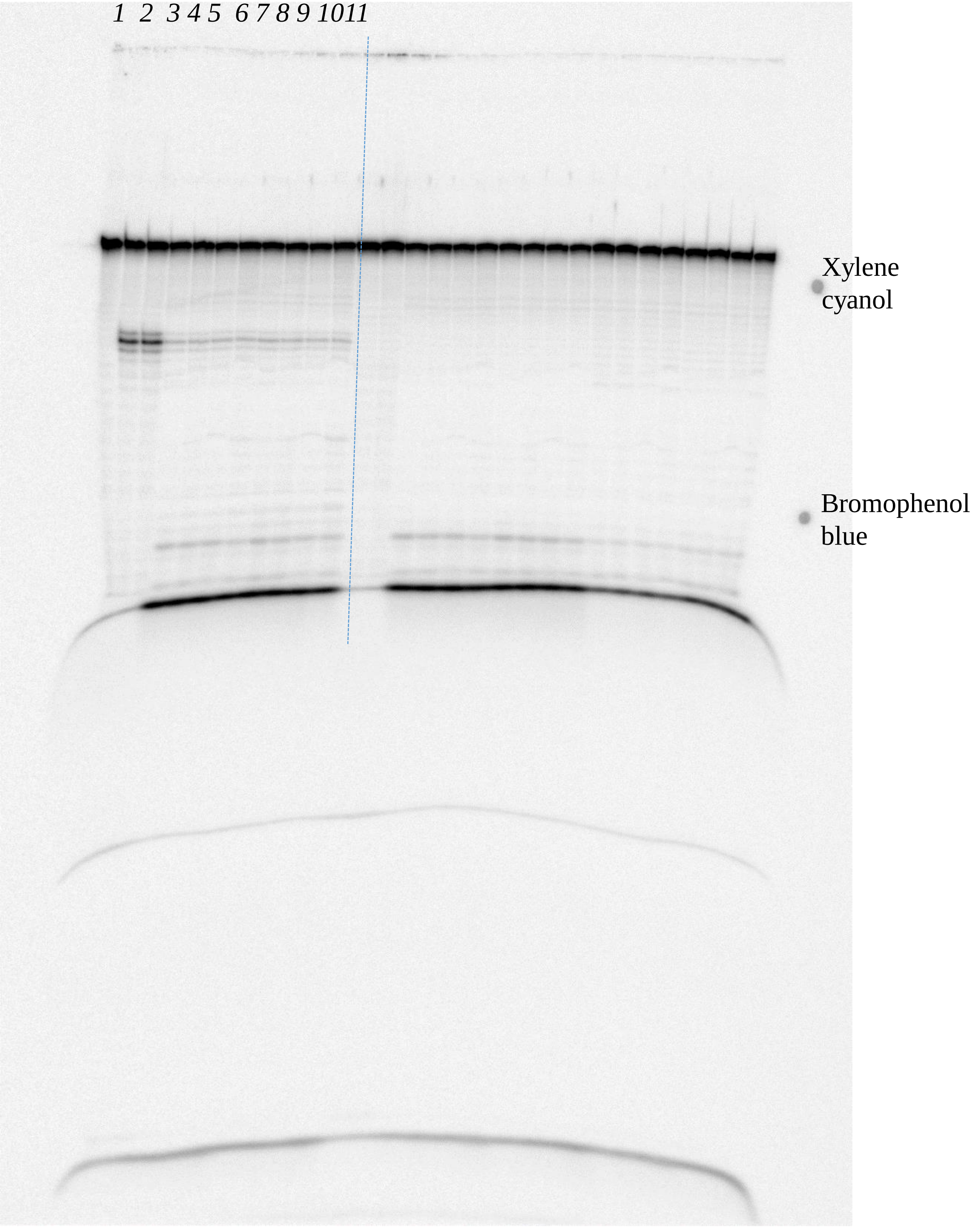

S7A Fig (bottom panel).
Numbering of the lanes corresponds to that in S7A Fig.

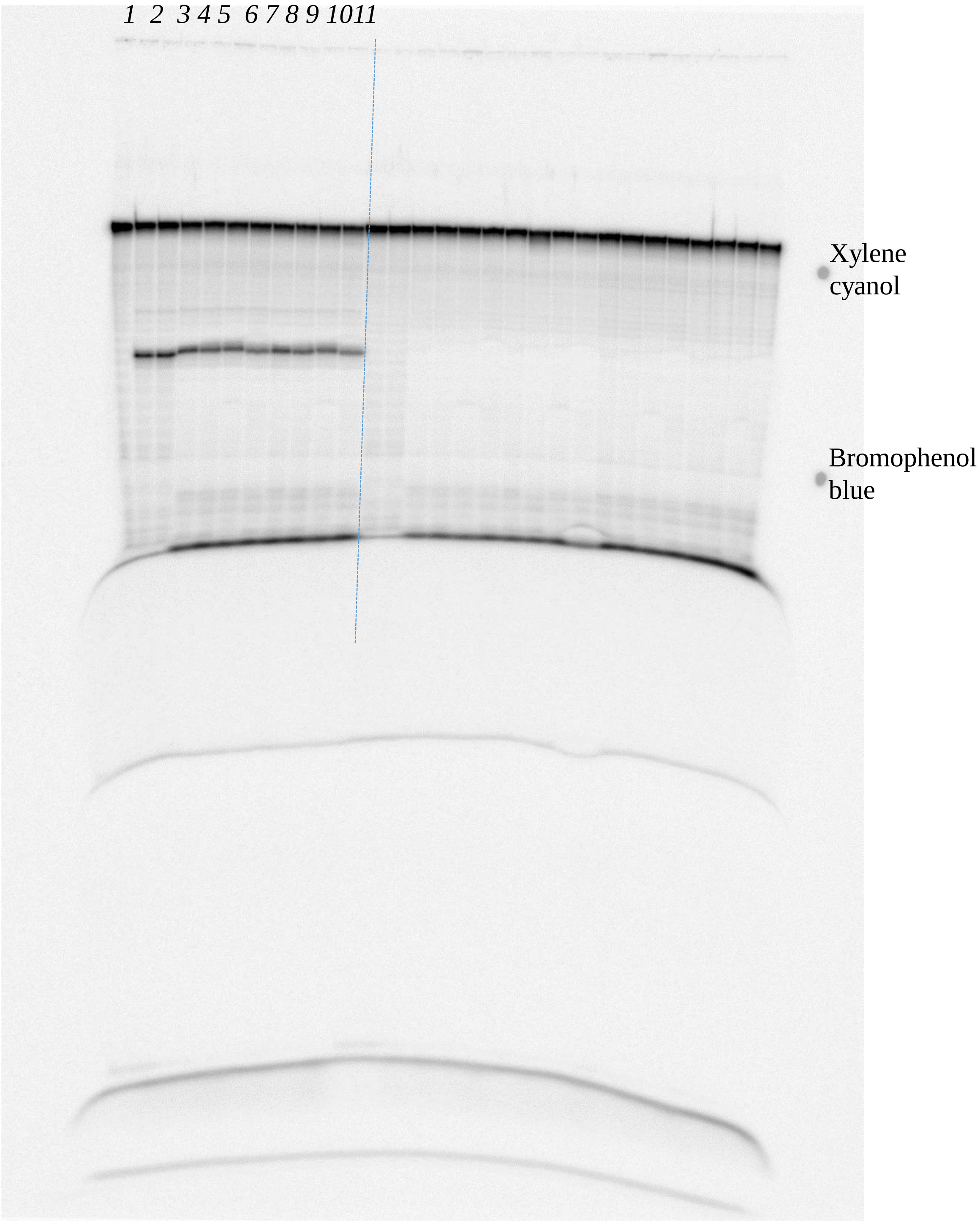

S8 Fig

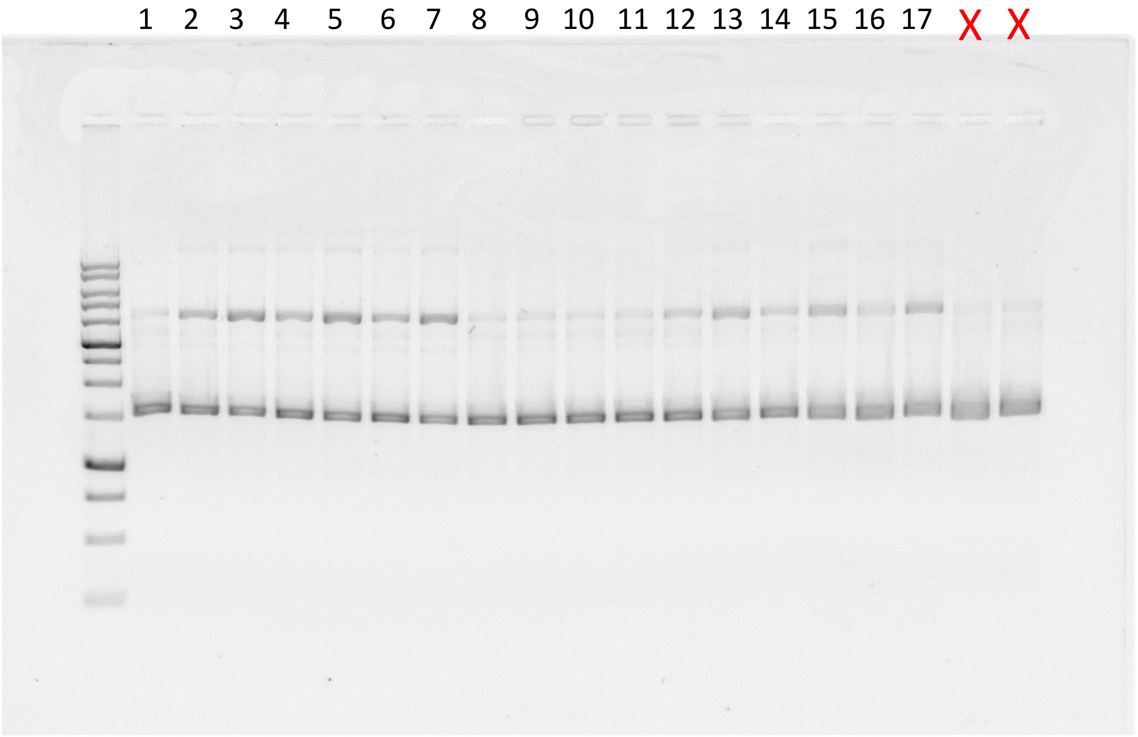

S9A and S9B Figs.
Numbering of the lanes corresponds to that in S9A and S9B Figs.

A

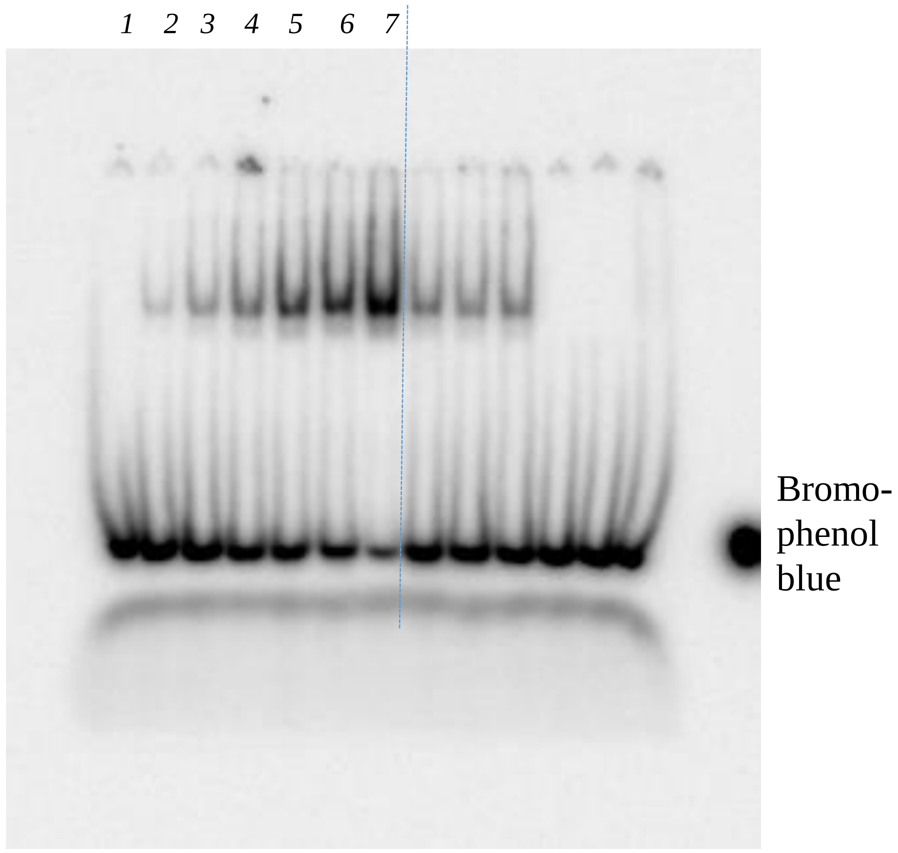

B

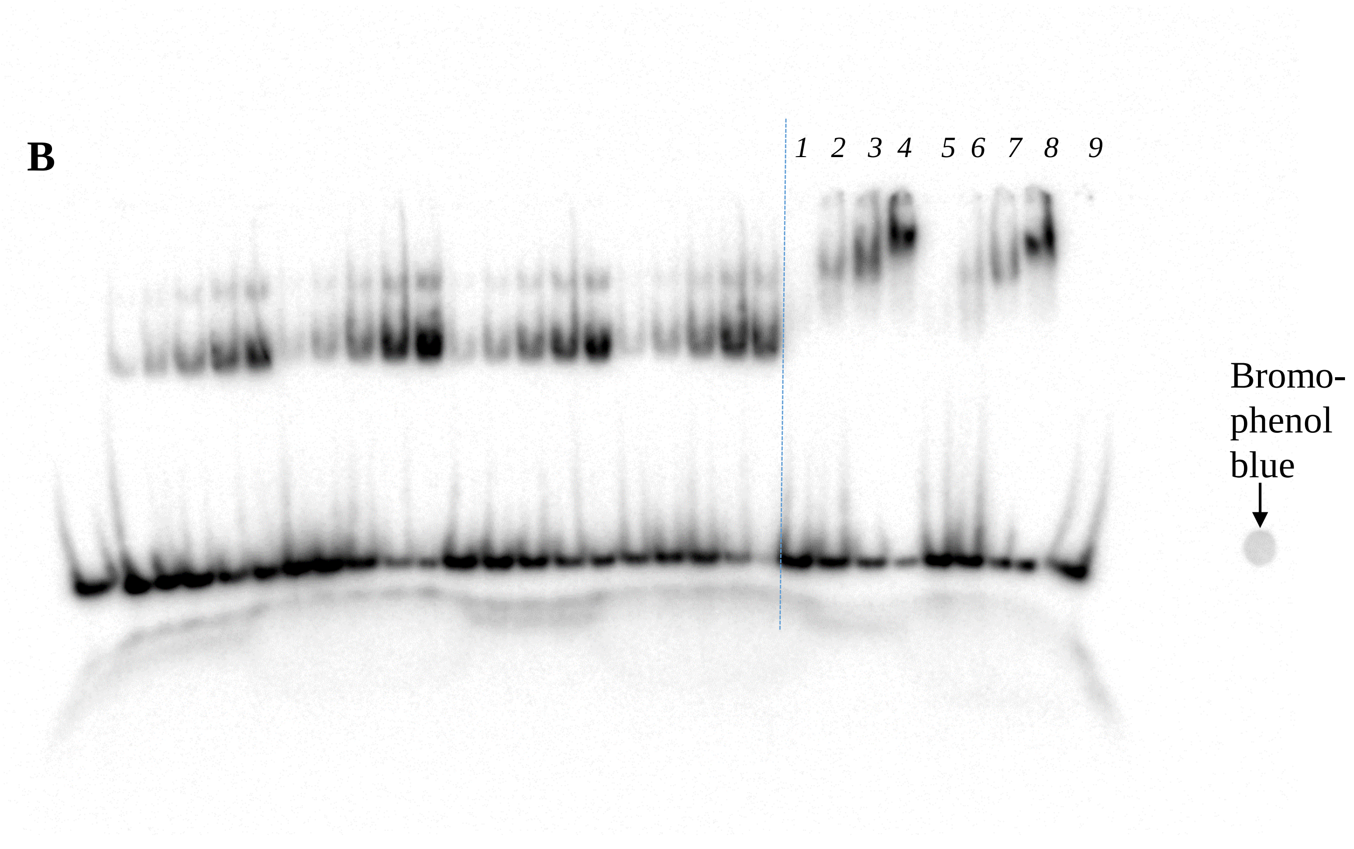

S9C Fig.
Numbering of the lanes corresponds to that in S9C Fig.

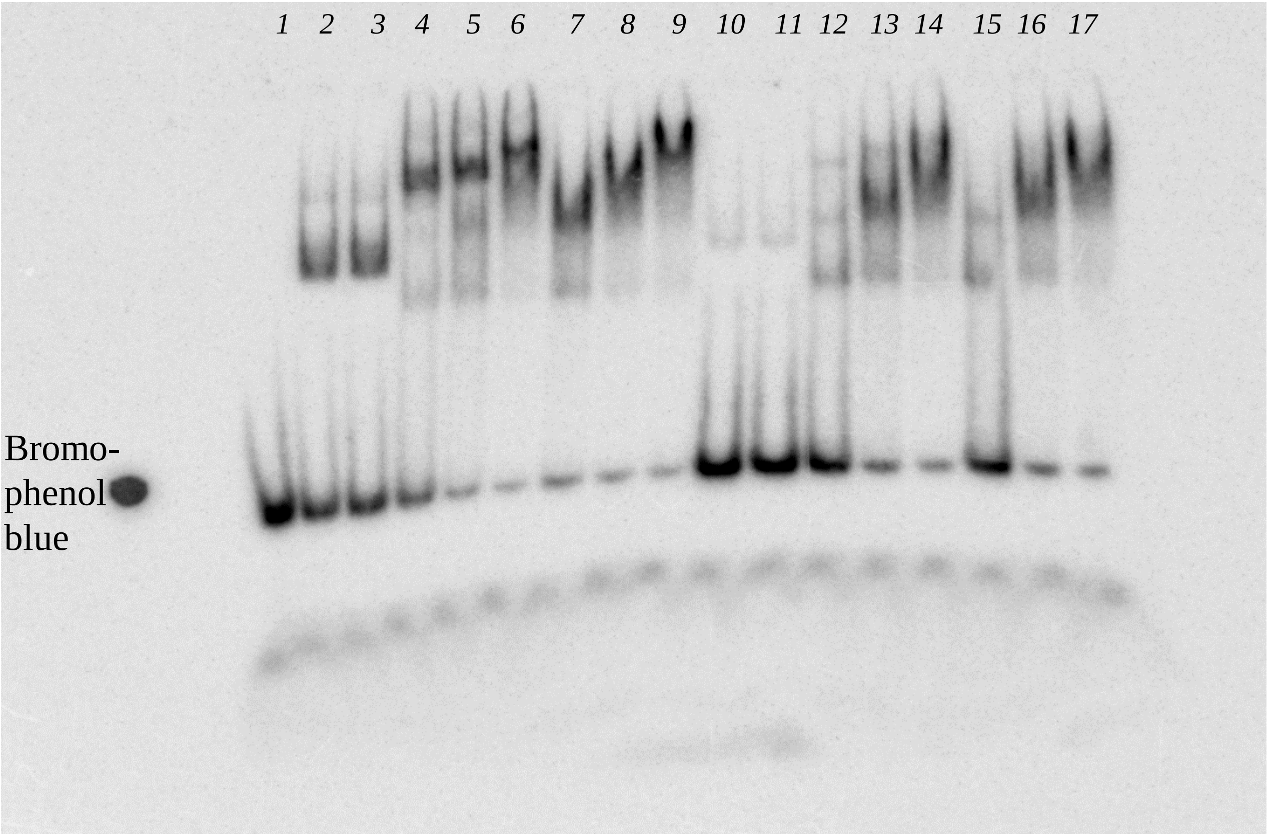

S10A Fig.
Numbering of the lanes corresponds to that in S10A Fig.

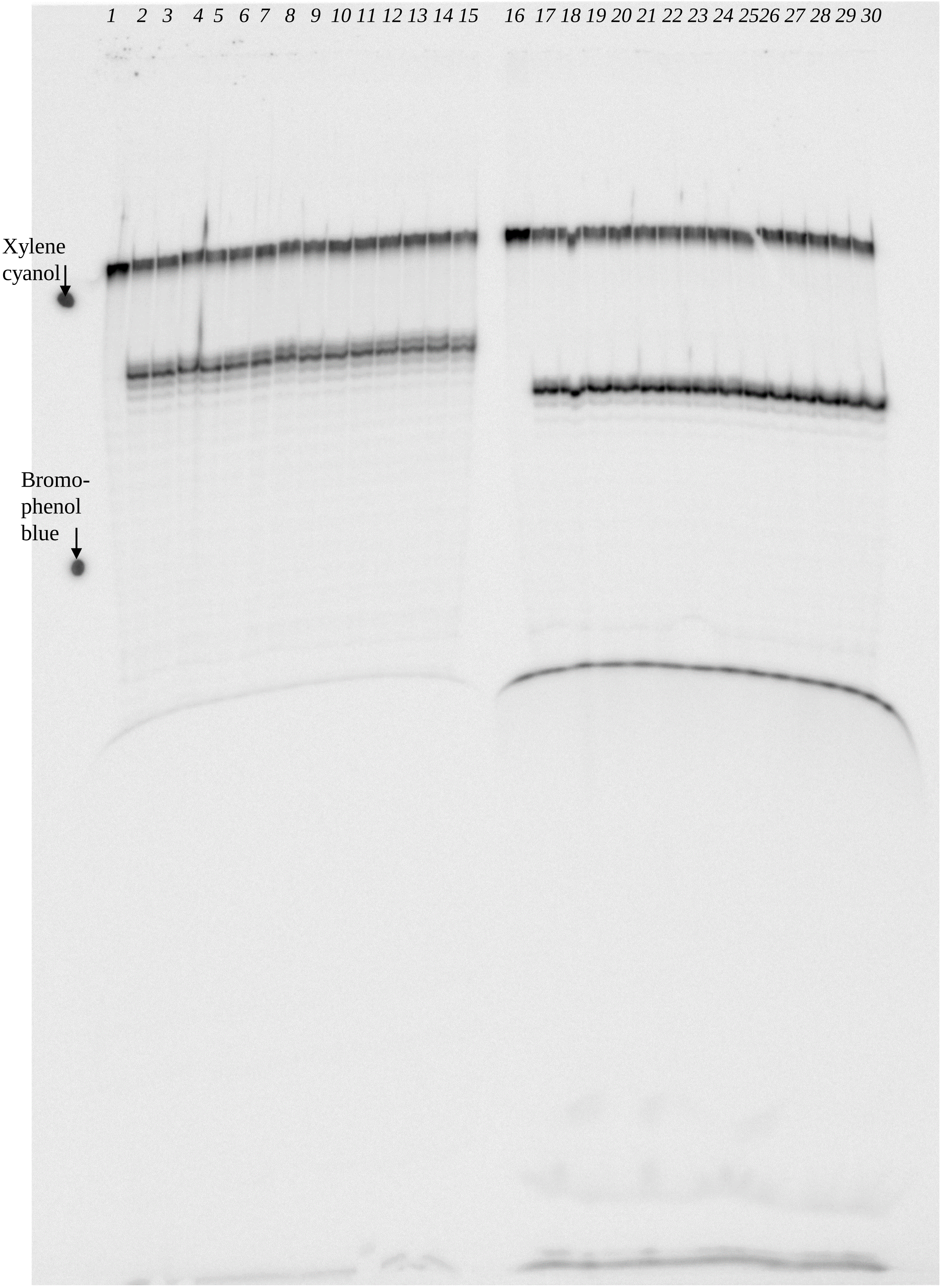

Supplement: S1 Raw images — (PDF) [file pone.0294683.s015.pdf]
