# Supplementary material for: Cas9 is mostly orthogonal to human systems of DNA break sensing and repair
Source: PLoS One. 2023 Nov 29;18(11):e0294683. doi: 10.1371/journal.pone.0294683 (PMC10686484; doi:10.1371/journal.pone.0294683)
Supplement: Attachment — Submitted filename: Reviewers answer.docx [file pone.0294683.s017.docx]

Dear Reviewers,

Thank you for considering the reviewed version of our manuscript, “Cas9 is mostly orthogonal to human systems of DNA break sensing and repair” (PONE-D-23-20117R1) by Maltseva *et al*. As Reviewer 1 did not raise further concerns, below please find our response to the remaining questions from Reviewer 2.

*Reviewer #2: In this revised version of the manuscript by Maltseva et al., the authors addressed some of my comments. Nevertheless, I was less convinced by their answers to my first two concerns, which were actually the most critical ones.*

*In their response to my first concern, the authors do not provide any data to support their claim that "we deem it unlikely that our results will be significantly influenced by the target DNA sequence." As such, I feel that the fact that the authors did not test other target sequence and sgRNA is a major weakness of the current work.*

We certainly agree that Cas9 can be targeted by sgRNA to different sequences. The main point of our initial response was that PARP1, PARP2, LigI, Ku70/80 and YB1 are non-sequence-specific proteins, and it can hardly be expected that they will interact with other sequences in a manner different from the Sp2 sequence. To check possible influence of the target DNA sequence on the activity of Cas9 and its interplay with key DNA repair factors, we performed additional experiments with another sgRNA and target DNA plasmid substrate.

The sequences of a new sgRNA_pMSH2_ and the previously used sgRNA_pLK1_ are presented below:

sgRNA_pMSH2_

5′‑AUCAAGUACAUGGGGCCGGCGUUUUAGAGCUAGAAAUAGCAAGUUAAAAUAAGGCUAGUCCGUUAUCAACUUGAAAAAGUGGCACCGAGUCGGUGCUUUU-3′

sgRNA_pLK1_

5′‑GGAUAACUCAAUUUGUAAAAAAGUUUUAGAGCUAGAAAUAGCAAGUUAAAAUAAGGCUAGUCCGUUAUCAACUUGAAAAAGUGGCACCGAGUCGGUGCUUUU-3′.

The DNA plasmid substrate corresponding to sgRNA_pMSH2_ was constructed based on a plasmid with inserted *hMSH2* gene (Addgene ID #16453).

First, we compared the Cas9-catalysed cleavage of both substrates (Fig. 1).

**Fig 1. Cleavage of two different DNA plasmid substrates by Cas9/sgRNA.** Cas9/sgRNA_pLK1_ (20 nM) or Cas9/sgRNA_pMSH2_ (20 nM) was incubated with pLK1 DNA (10 ng/µl) or pMSH2 DNA (10 ng/µl), respectively. S, substrate (supercoiled plasmid pMSH2 or pLK1); P1, SSB containing product (nicked plasmids); P2, DSB-containing product (linear plasmids). The curves show the accumulation of P1+P2 products under the indicated conditions. The sizes of DNA markers are shown next to the gel images.

The activity of Cas9 in cleavage of the pMSH2 DNA was revealed to be 3-fold lower compared the pLK1 DNA. The difference most likely results from the higher GC content of the protospacer seed region of the former substrate.

Next, we explored the influence of several DNA binding proteins on the of Cas9-catalysed cleavage of the pMSH2 DNA (Fig. 2). No significant change in the efficiency of pMSH2 DNA cleavage was detected in the presence of PARP1/2 at a high excess over Cas9, regardless of the presence of NAD^+^. Similarly, no modulating effects produced by either Ku70/80 or RPA could be observed. Thus, the ability of Cas9 to efficiently shield DSBs and SSBs from their sensors does not depend on the targeted DNA sequence.

**Fig 2. Cleavage of the pMSH2 plasmid substrate by Cas9/sgRNA_pMSH2_ in the presence of different proteins.** Cas9/sgRNA_pMSH2_ (20 nM) was incubated with pMSH2 DNA (10 ng/µl) in the absence and presence of PARP1 (100 nM) or PARP2 (500 nM) without or with 500 μM NAD^+^, Ku70/80 (250 nM), and RPA (500 nM) for 15 min at 37°C. S, substrate (supercoiled pMSH2 plasmid); P1, SSB containing product (nicked plasmid); P2, DSB-containing product (linear plasmid). The bar chart shows the extent of pMSH2 DNA cleavage in the presence of the indicated proteins.

All new data, including the supplementary figure S10, have been added to the revised text (p. 15, lines 353-359). According to this the numbering of supplementary figures and the supplementary figure references in main text have been changed.

*Regarding my second point, unless I missed something, I still believe that the current competition experiments using dsDNA to test whether PARP1 or Ku could displace Cas9 and then affect its catalytic activity are difficult to interpret. Indeed both PARP1 and Ku will probably bind to the DNA ends with high affinity and therefore have no reason to compete with Cas9 since they bind to different locations along the dsDNA. This limitation is actually even mentioned by the authors themselves when they write that "To exclude binding of PARPs to the DNA duplex blunt ends, we further utilized the supercoiled pLK1 plasmid as a DNA ligand." (line 162). Unfortunately, the use of the pLK1 plasmid is not completely appropriate since it does not allow to assess protein binding by gel shift. So overall I still feel that with the current samples (dsDNA and pLK1 plasmid) the authors are not able to adequately address the question of the competition between Cas9 and PARP1 or Ku.*

We surmise that we and the Reviewer are actually talking about the same thing but our narrative was too confusing. The purpose of these experiments was exactly to see whether PARPs’ apparent lack of competition with Cas9 observed when following the activity is due to binding to different parts of DNA. Essentially, the ds oligo experiments is relevant to the lack of competition on dsDNA1/2, presented in Fig. 1A,B, whereas the plasmid experiment is relevant to the lack of competition on pLK1, presented in Fig. 1C,D. With the ds oligo, the lack of binding competition is clearly seen from the same PARP concentration dependence of band shift without (Supplementary Fig. S5) or with Cas9 (Fig. 2A,B). The plasmid experiment, on the other hand, tells us that even at the PARP:plasmid molar ratio of ~60:1 (200 nM) PARP1 molecules are bound along DNA in an amount significant enough to shift it (Supplementary Fig. S5, refer to lanes 12 and 13) but this has little positive or negative effect on Cas9 binding with or without sgRNA (Fig. 2C).

The reviewer’s suggestion to use alternative DNA structures does not seem particularly useful, since at least PARP1 and Ku70/80 are known to bind various non-canonical nucleic acid structures, including hairpin loops [Laspata et al., J. Mol. Biol., 2023, 20:168207. doi: 10.1016/j.jmb.2023.168207; Arosio et al., J. Biol. Chem., 2002, 277:9741-8. doi: 10.1074/jbc.M111916200]. Thus, such substrates would not help to eliminate the competition at DNA duplex ends, as a circular plasmid does.

The revised version of the manuscript has been approved by all authors.

Sincerely yours,

Olga Lavrik and co-authors
